# VEGF dose regulates vascular stabilization through Semaphorin3A and the Neuropilin-1^+^ monocyte/TGF-β1 paracrine axis

**DOI:** 10.15252/emmm.201405003

**Published:** 2015-09-07

**Authors:** Elena Groppa, Sime Brkic, Emmanuela Bovo, Silvia Reginato, Veronica Sacchi, Nunzia Di Maggio, Manuele G Muraro, Diego Calabrese, Michael Heberer, Roberto Gianni-Barrera, Andrea Banfi

**Affiliations:** 1 Department of Biomedicine, University of BaselBasel, Switzerland; 2 Department of Surgery, Basel University HospitalBasel, Switzerland

**Keywords:** monocyte; TGF-β1, semaphorin3A, vascular stabilization, VEGF

## Abstract

VEGF is widely investigated for therapeutic angiogenesis, but while short-term delivery is desirable for safety, it is insufficient for new vessel persistence, jeopardizing efficacy. Here, we investigated whether and how VEGF dose regulates nascent vessel stabilization, to identify novel therapeutic targets. Monoclonal populations of transduced myoblasts were used to homogeneously express specific VEGF doses in SCID mouse muscles. VEGF was abrogated after 10 and 17 days by Aflibercept treatment. Vascular stabilization was fastest with low VEGF, but delayed or prevented by higher doses, without affecting pericyte coverage. Rather, VEGF dose-dependently inhibited endothelial Semaphorin3A expression, thereby impairing recruitment of Neuropilin-1-expressing monocytes (NEM), TGF-β1 production and endothelial SMAD2/3 activation. TGF-β1 further initiated a feedback loop stimulating endothelial Semaphorin3A expression, thereby amplifying the stabilizing signals. Blocking experiments showed that NEM recruitment required endogenous Semaphorin3A and that TGF-β1 was necessary to start the Semaphorin3A/NEM axis. Conversely, Semaphorin3A treatment promoted NEM recruitment and vessel stabilization despite high VEGF doses or transient adenoviral delivery. Therefore, VEGF inhibits the endothelial Semaphorin3A/NEM/TGF-β1 paracrine axis and Semaphorin3A treatment accelerates stabilization of VEGF-induced angiogenesis.

## Introduction

Atherosclerotic coronary and peripheral artery diseases remain major causes of morbidity and mortality, despite optimal surgical and medical therapies (Norgren *et al*, 2007; Go *et al*, 2014). An attractive strategy to fill this unmet clinical need is therapeutic angiogenesis, that is the growth of normal, stable, and functional blood vessels by delivering specific factors to ischemic tissues, whereby the robust expansion of microvascular networks promotes the growth of collateral arteries (arteriogenesis) through increased shear stress and retrograde signals along the vessel walls via intercellular gap junctions (Rissanen *et al*, 2005; Pries *et al*, 2010). Vascular endothelial growth factor-A (VEGF) is the master regulator of vascular growth in both development and postnatal life, and it represents the major molecular target for therapeutic angiogenesis (Giacca & Zacchigna, 2012). However, VEGF therapeutic potential depends on both its dose and duration of expression. In fact, uncontrolled and sustained VEGF expression, achieved by a variety of gene therapy vectors (Isner *et al*, 1996; Pettersson *et al*, 2000; Schwarz *et al*, 2000; Sundberg *et al*, 2001; Zacchigna *et al*, 2007; Karvinen *et al*, 2011) or cell-based approaches (Springer *et al*, 1998; Lee *et al*, 2000), can cause the growth of aberrant vascular structures and angioma-like tumors in both normal and ischemic tissues. On the other hand, *in vivo* inducible systems (Dor *et al*, 2002; Tafuro *et al*, 2009) and treatment with the blocking receptor-body VEGF-Trap_R1R2_ (Aflibercept) (Ozawa *et al*, 2004) showed that VEGF expression shorter than about 4 weeks is insufficient to stabilize normal newly induced vessels, leading to their regression after stimulus cessation, although dose-dependent kinetics were not investigated. Therefore, there is a need to identify molecular targets to accelerate vascular stabilization despite short-term VEGF delivery.

The best understood mechanism leading to stabilization of newly induced vessels is maturation, that is their association with pericytes, which suppress endothelial proliferation and provide survival signals such as Angiopoietin-1 (Ang-1) and low levels of localized VEGF expression (Darland *et al*, 2003; Carmeliet & Jain, 2011). While maturation is a morphological feature of new vessels (pericyte association), stabilization is the functional property of persisting independently of further VEGF stimulation (Dor *et al*, 2002; Potente *et al*, 2011) and is a therapeutically relevant property defining the minimum necessary duration of VEGF delivery to achieve a persistent increase in vascularity. Different populations of bone marrow (BM)-derived mononuclear cells also are recruited to the sites of VEGF-induced angiogenesis in adult tissues, where they do not incorporate into the newly formed vessels (Ziegelhoeffer *et al*, 2004; Zentilin *et al*, 2006), but exert pro-angiogenic effects by secreting paracrine factors (Grunewald *et al*, 2006; Korpisalo *et al*, 2008). In particular, a specific population of monocytes co-expressing CD11b and the VEGF co-receptor Neuropilin-1 (NRP1), and named therefore neuropilin-expressing monocytes (NEM), do not stimulate endothelial proliferation and vascular growth, but specifically favor pericyte and smooth muscle cell recruitment during VEGF-induced angiogenesis by secreting transforming growth factor-β (TGF-β) and platelet-derived growth factor-BB (PDGF-BB) (Zacchigna *et al*, 2008), leading also to normalization of tumor vessels and inhibiting tumor growth (Carrer *et al*, 2012). However, it is unknown whether monocytes may also directly regulate the acquisition of VEGF-independence by newly induced endothelial structures.

Here, we found that increasing doses of VEGF, in a therapeutically relevant range, negatively regulated vascular stabilization in a dose-dependent fashion, without affecting pericyte recruitment and maturation, but rather by directly inhibiting endothelial expression of the NRP1 ligand Semaphorin3A (Sema3A) and the NEM/TGF-β1 paracrine axis. Further, treatment with recombinant Sema3A counteracted the effects of increasing VEGF dose and accelerated stabilization of VEGF-induced angiogenesis in the therapeutic target tissue of skeletal muscle, enabling vessel persistence despite transient VEGF delivery by adenoviral vectors.

## Results

### Vascular stabilization is progressively impaired by increasing VEGF doses

**Figure 1:**
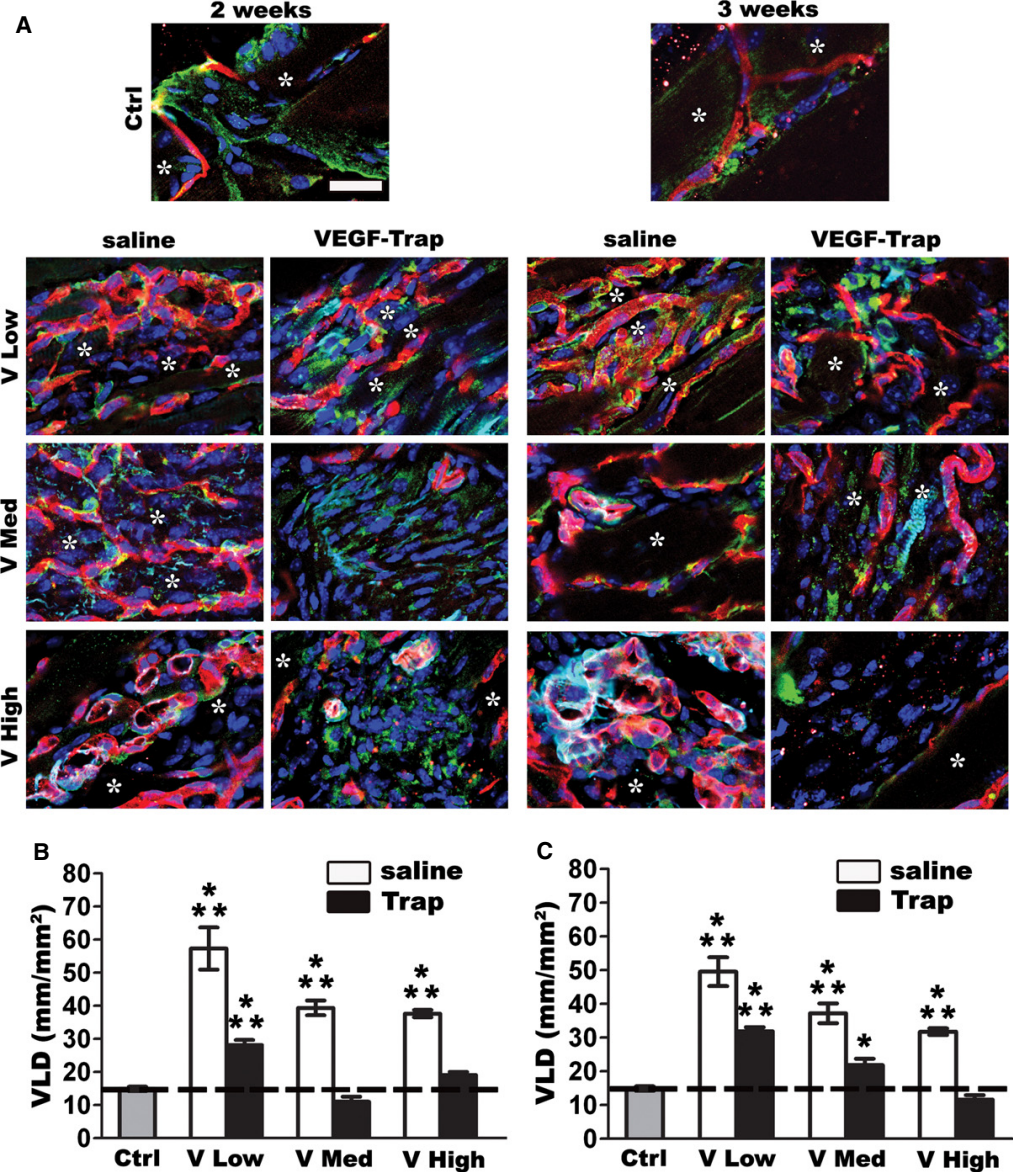
VEGF impairs vascular stabilization despite similar pericyte coverage A Immunofluorescence staining of endothelium (CD31, in red), pericytes (NG2, in green), smooth muscle cells (α-SMA, in cyan), and nuclei (DAPI, in blue) on frozen sections of limb muscles injected with myoblast clones expressing different VEGF levels (V Low, V Med, and V High, respectively) and treated with VEGF-Trap or saline after 10 and 17 days. Analysis was performed after 4 days of treatment (2 and 3 weeks, respectively). All normal vessels induced by low and medium VEGF doses displayed a similar coverage by normal pericytes, whereas aberrant structures induced by high VEGF were covered with α-SMA-positive smooth muscle cells. Asterisks indicate muscle fibers, which were mostly sectioned in longitudinal orientation, although some cross sections are visible depending on the orientation of muscle bundles. Scale bar = 25 μm.
B, C Quantification of vessel length density (VLD) on the same samples showed that stabilization started already by 10 days for vessels induced by low VEGF, but not until 17 days for those induced by medium VEGF, despite similar pericyte coverage, while aberrant structures induced by high VEGF always regressed. Data represent the mean ± SEM of individual images (*n*) acquired from three muscles/group; 2 weeks (B): V Low saline, *n* = 9; V Med saline, *n* = 13; V High saline, *n* = 8; V Low TRAP,*n* = 28; V Med TRAP,*n* = 22; V High TRAP,*n* = 30; 3 weeks (C): V Low saline, *n* = 9; V Med saline, *n* = 11; V High saline, *n* = 19; V Low TRAP,*n* = 43; V Med TRAP,*n* = 14; V High TRAP,*n* = 19; *P < 0.05 and ***P < 0.001 (all versus Ctrl) by one-way ANOVA with Bonferroni multiple comparisons test; 2 weeks (B): V Low TRAP versus Ctrl *P* < 0.0001; 3 weeks (C): V Low TRAP versus Ctrl *P* < 0.0001; V Med TRAP versus Ctrl *P* = 0.0158. Source data are available online for this figure.

As we previously found that the therapeutic window of VEGF overexpression is controlled by the dose of VEGF localized in the microenvironment around each expressing cell (Ozawa *et al*, 2004; von Degenfeld *et al*, 2006), to rigorously determine the role of VEGF dose on the vascular stabilization kinetics we took advantage of a unique and well-characterized platform, based on monoclonal populations of retrovirally transduced mouse myoblasts that express specific VEGF_164_ doses, thereby ensuring homogeneous microenvironmental levels (Ozawa *et al*, 2004; Misteli *et al*, 2010). We selected three clones expressing increasing VEGF levels *in vitro*, previously shown to induce either normal (low and medium) or aberrant angiogenesis (high): V low = 11.0 ± 0.4 ng/10^6^ cells/day, V med = 61.0 ± 2.9 ng/10^6^ cells/day, and V high = 133.2 ± 9.7 ng/10^6^ cells/day. Populations were implanted into leg skeletal muscles of adult SCID mice to avoid immunologic response to myoblasts expressing xenogeneic LacZ protein (Misteli *et al*, 2010). All clones engrafted with similar efficiency over the first week after implantation, as determined by qRT–PCR quantification of the genomically integrated β-gal retroviral copies (Appendix Fig S1). Vessel stabilization was determined as the fraction of newly induced vessels that was capable of surviving independently of VEGF signaling, by quantifying the persisting vessel length density (VLD) after systemic treatment with VEGF-Trap_R1R2_ (Aflibercept), a potent receptor-body shown to completely abrogate VEGF signaling *in vivo* (Holash *et al*, 2002), or saline control. The same VEGF-Trap dose was previously shown to efficiently abrogate all VEGF levels used here with the same myoblast-based platform, whereas it did not affect pre-existing vessels in the absence of VEGF overexpression (Ozawa *et al*, 2004). As expected, after 2 and 3 weeks, both low and medium VEGF levels yielded a network of homogeneous capillaries, whereas high VEGF led to aberrant angioma-like structures (Fig 1A). Muscle areas remote from the site of myoblast implantation (as determined by X-gal staining) in saline-treated mice were used as controls. The mean VLD of pre-existing muscle capillaries in these areas was 15 ± 0.7 mm/mm^2^ and the VLD increase above this value represents the amount of newly induced vessels. Trap treatment was started 4 days before tissue harvest. Therefore, the effects observed at the analysis time points of 2 and 3 weeks reflect the stabilization achieved at the start of treatment, 10 and 17 days after cell injection, respectively. After 10 days, 31 ± 3.6% of the vessels induced by low VEGF were already VEGF-independent, whereas similarly normal vessels induced by medium VEGF regressed completely, as well as the aberrant structures induced by high VEGF, reducing VLD to a similar value as the pre-existing capillary networks in control areas (Fig 1A and B). By 17 days, the fraction of stabilized new vessels induced by low VEGF increased to 49 ± 3.5%, while 31 ± 8.3% became VEGF-independent with medium VEGF levels (Fig 1A and C). Aberrant vascular structures induced by high VEGF were still completely sensitive to VEGF deprivation. Therefore, increasing VEGF levels, within the range that induces only normal angiogenesis, dose-dependently impaired the stabilization of newly induced capillaries.

### Stabilization rate does not correlate with differential pericyte coverage or vascular perfusion

Both pericyte recruitment and establishment of functional flow have been shown to provide crucial signals for the stabilization of nascent vascular structures (Potente *et al*, 2011). Pericyte coverage of newly induced vessels was quantified 2 weeks after implantation of the different clones in hind-limb muscles by measuring their maturation index, that is, the ratio of the NG2-positive/CD31-positive areas after immunofluorescence staining. As shown in Fig 1A, the normal capillaries induced by both low and medium VEGF were tightly associated with NG2-positive/α-SMA-negative pericytes, with a similar maturation index (Vlow = 0.4 ± 0.02 and Vmed = 0.4 ± 0.06, *P* = N.S.). As previously described (Ozawa *et al*, 2004), aberrant structures induced by high VEGF were not covered by pericytes, but rather by NG2-negative/α-SMA-positive smooth muscle cells (Fig 1A), which have a different biological function, providing structural support but not regulatory signals to the endothelium, and therefore their maturation index could not be quantified.

**Figure EV1:**
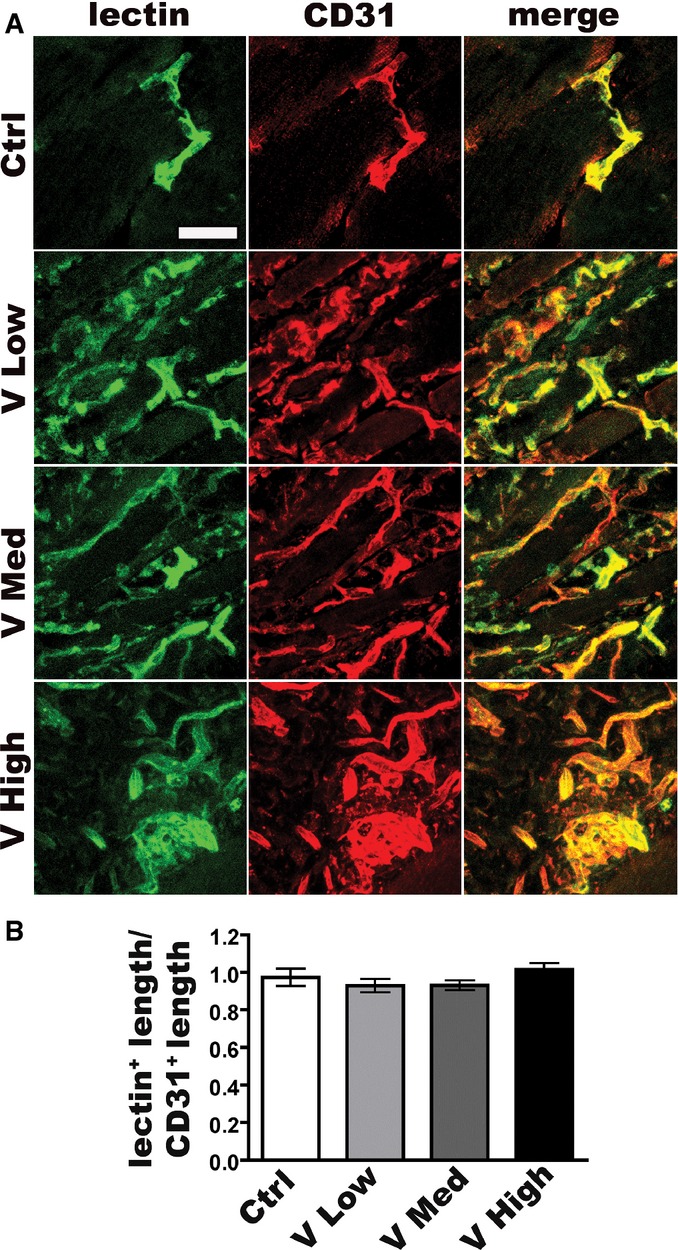
Vessels induced by different VEGF doses are similarly perfused Mice received intravenous injections of FITC-lectin 2 weeks after implantation of myoblast clones expressing different VEGF levels. Frozen sections of limb muscles were immunostained for CD31 (endothelium, in red) and perfused structures were visualized by FITC-lectin co-localization (in green). Scale bar = 50 μm.
Quantification of the perfusion index (lectin-positive vessel length/CD31-positive vessel length) showed that both normal capillaries and aberrant structures induced by the different VEGF doses were similarly perfused. Data represent the mean ± SEM of individual images (*n*) acquired from three muscles/group: Ctrl, *n* = 3; V Low, *n* = 8; V Med, *n* = 8; V High, *n* = 7. Data were subjected to Kruskal–Wallis analysis with Dunn’s multiple comparisons test and no significant differences were detected. Source data are available online for this figure.

The establishment of functional blood flow in newly induced vascular structures was assessed by intravenous injection of fluorescently labeled tomato lectin, which binds to the luminal endothelial surface of vessels only if they are perfused by the systemic circulation (von Degenfeld *et al*, 2006). Both normal vessels and aberrant structures induced by all VEGF doses were stained by lectin and therefore functionally perfused (Fig EV1A). Further, quantification of the ratio between lectin-positive and CD31-positive vessel lengths did not show any significant difference among the groups (Fig EV1B) and very few non-perfused endothelial structures were visible in every condition with similar frequency. These results suggest that neither differential pericyte recruitment nor establishment of functional flow were responsible for the different stabilization kinetics of normal vessels induced by low and medium VEGF doses.

### TGF-β1 and Sema3A are downregulated in tissues exposed to increasing VEGF doses

**Figure 2:**
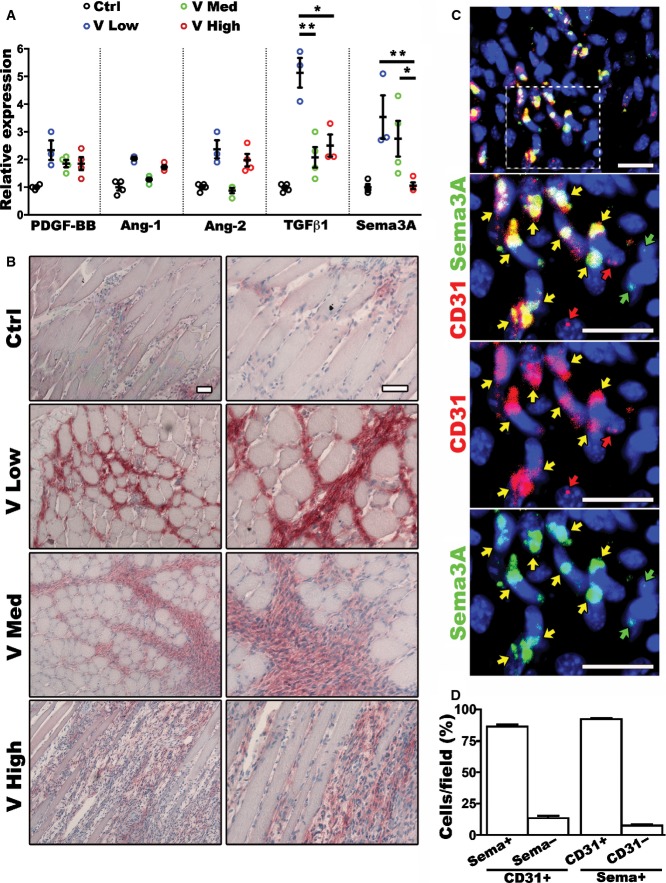
TGF-β1 and Sema3A are downregulated *in vivo* by increasing VEGF doses Muscles were harvested 7 days after implantation of V Low, V Med, and V High clones or control cells (Ctrl). Gene expression of *Pdgfb*,*Ang1*,*Ang2*,*Tgfb1*, and *Sema3a* was quantified by qRT-PCR and expressed as fold-change versus control muscles. Data represent individual values, with mean ± SEM (*n* = 3 or 4, as indicated); *P < 0.05 and **P < 0.01 by one-way ANOVA with Bonferroni multiple comparisons test, after data normalization by logarithmic transformation. TGF-β1: V Low versus V Med *P* = 0.0047; V Low versus V High *P* = 0.0379; Sema3A: V Low versus V High *P* = 0.0042; V Med versus V High *P* = 0.0179.
Immunohistochemistry for Sema3A on frozen muscle sections confirmed a decreasing protein expression in the areas implanted with myoblasts producing increasing VEGF doses (*n* = 4). The right panels represent higher-magnification views of the left panels, which encompass the majority of the implantation sites. Scale bar = 50 μm.
*In situ* hybridization for Sema3A (green) and CD31 (red) mRNA, with nuclei staining by DAPI, on frozen muscle sections. The three lower panels represent a higher-magnification of the area in the white square in the top panel, as a merged image and as individual channels: arrows indicate cells expressing both transcripts (yellow), only Sema3A (green), or only CD31 (red). Scale bar = 30 μm.
Quantification of the percentage of endothelial cells (CD31^+^) that express Sema3A (left group) and of Sema3A-expressing cells that are endothelial (CD31^+^, right group). Data represent the mean ± SEM of 12 individual fields of view from three independent muscles (15–94 nuclei/image, 642 total nuclei). No statistics was applied, as data represent complementary sets. Source data are available online for this figure.

To investigate the mechanism by which increasing VEGF doses impaired the stabilization of similarly normal and pericyte-covered capillaries, we quantified the expression of the main vascular maturation and remodeling factors (PDGF-BB, Ang-1, Ang-2, and TGF-β1) and of Sema3A. Sema3A is a secreted axon-guidance protein that can be expressed by endothelial cells and, through binding to NRP1, can recruit neuropilin1-expressing monocytes (NEM) (Zacchigna *et al*, 2008), although its role in the stabilization of VEGF-induced angiogenesis is unknown. Muscles were injected with the 3 VEGF-expressing clones or control myoblasts and, since the differences in vascular stabilization were detected already with VEGF-Trap treatment starting 10 days after cell implantation, gene expression was measured at day 7 in order to detect the most relevant differences for the stabilization process. As shown in Fig 2A, *Pdgfbb*,*Ang1*, and *Ang2* were moderately upregulated in tissues exposed to low VEGF, but their expression patterns with increasing VEGF doses did not correlate with the observed decreasing trend in stabilization rates. On the other hand, both *Sema3a* and *Tgfb1* expression were robustly increased 4- to 5-fold compared to control levels with low VEGF and were significantly downregulated with higher VEGF doses. Gene expression data were confirmed by immunostaining for Sema3A protein on tissue sections, which showed a clear and progressive reduction in Sema3A abundance down to control levels in the areas of active angiogenesis by increasing VEGF doses (Fig 2B). Further, the different myoblast populations all expressed similar levels of *Sema3a* that were unrelated to the amount of VEGF, thereby excluding that the VEGF-expressing cells may be the source of the different Sema3A amounts observed *in vivo* (Appendix Fig S2).

In order to verify whether Sema3A was produced by endothelium or other populations, fluorescence *in situ* hybridization (FISH) was performed to co-detect the mRNA for *cd31* (marking endothelium) and *Sema3a* on frozen sections of muscles implanted with V low myoblasts at the 7-day time point, that is the condition displaying the highest Sema3A expression. Areas of myoblast implantation were identified by X-gal staining on adjacent serial sections and, as shown in Fig 2C, the green signal from the *Sema3a* transcript was detected mostly in cells that also expressed *cd31* (yellow arrows), whereas only rare cells were positive for either *Sema3a* or *cd31* alone (green and red arrows, respectively). Quantification of the number of cells expressing either or both transcripts showed that 86.4 ± 1.7% of the endothelial cells expressed Sema3A and that 92.3 ± 0.9% of the Sema3A-expressing cells was endothelial (Fig 2D). Therefore, endothelium was the source of the greatest part of Sema3A produced in the areas of active angiogenesis.

### Increasing VEGF doses impair *in vivo* endothelial Sema3A expression and NEM recruitment

**Figure 3:**
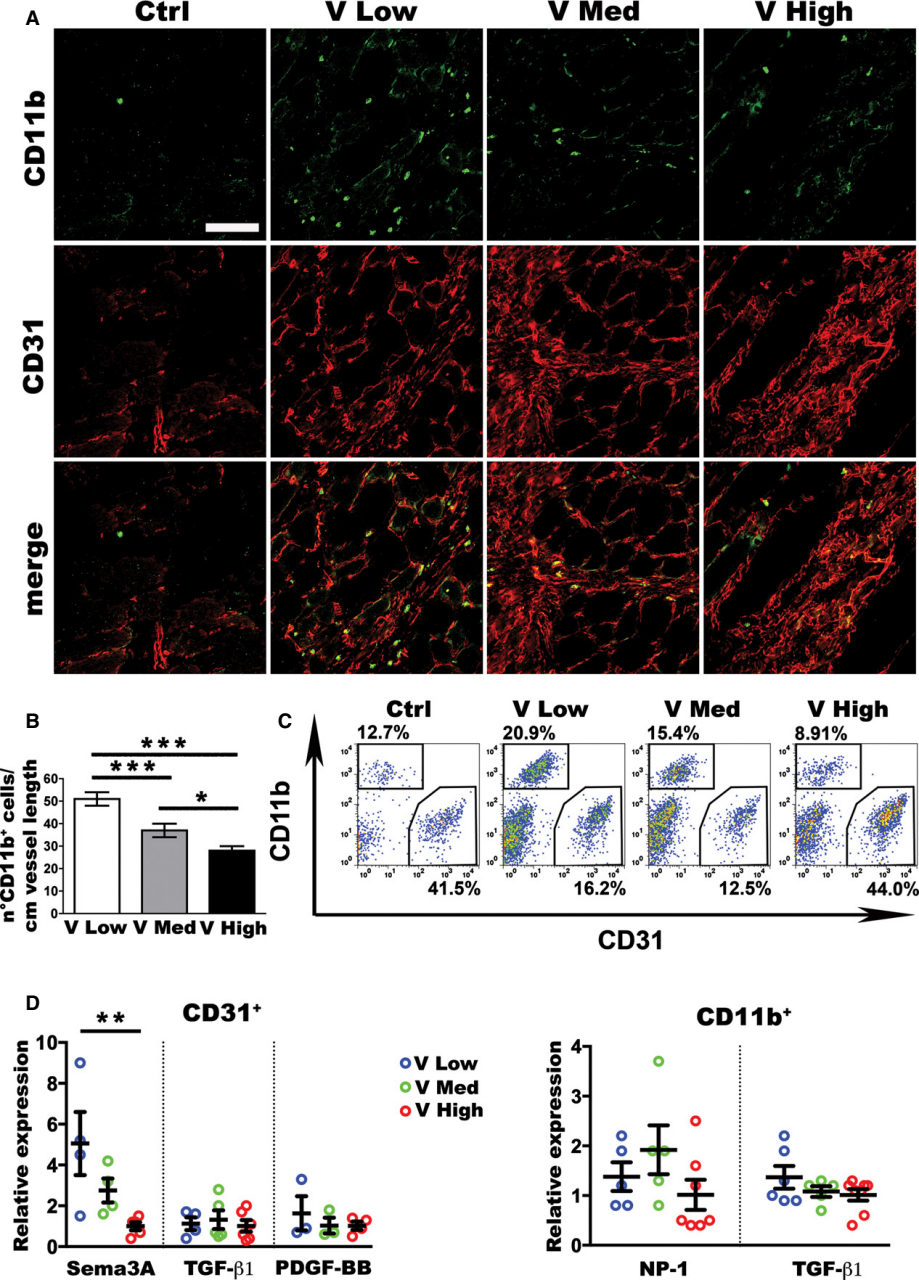
Increasing VEGF doses inhibit NEM recruitment and endothelial Sema3A expression Immunofluorescence staining of endothelial cells (CD31, in red) and NEM (CD11b, in green) on cryosections of limb muscles 1 week after injection with V Low, V Med, and V High myoblast clones. Scale bar = 100 μm.
Quantification of the number of NEM/cm of vessel length in sites of new angiogenesis by increasing VEGF doses. Data represent the mean ± SEM of individual images (*n*) acquired from three muscles/group: V Low, *n* = 48; V Med, *n* = 39; V High, *n* = 94; *P < 0.05 and ***P < 0.001 by one-way ANOVA with Bonferroni multiple comparisons test: V Low versus V Med *P* = 0.0008; V Low versus V High *P* < 0.0001; V Med versus V High *P* = 0.0304.
Endothelial cells (CD31^+^ quadrant) and NEM (CD11b^+^ quadrant) were sorted by FACS from muscles 1 week after injection with the same myoblast clones.
Relative gene expression was quantified in the *ex vivo* FACS-purified endothelial cells (CD31^+^) and NEM (CD11b^+^) and expressed as fold-change versus the V High group. Data represent individual values, with mean ± SEM (*n* = 3–7, as indicated); **P < 0.01 by one-way ANOVA with Bonferroni multiple comparisons test, after data normalization by logarithmic transformation; Sema3A: V Low versus V High *P* = 0.0157. Source data are available online for this figure.

**Figure EV2:**
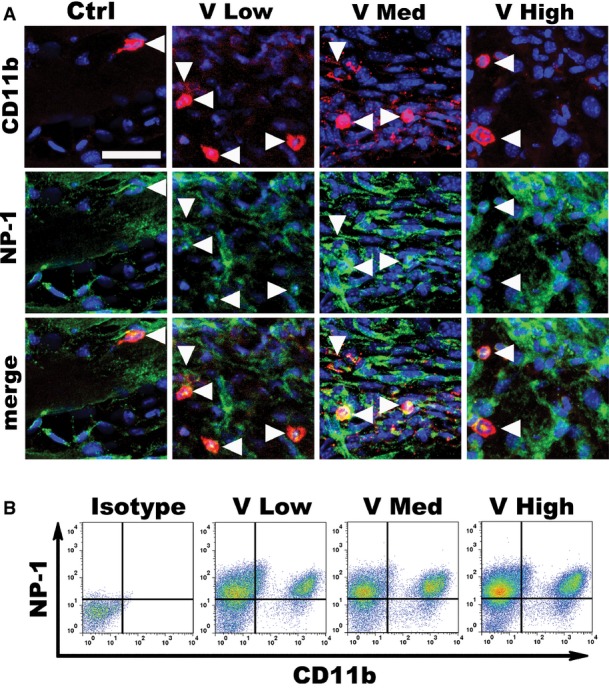
Mononuclear cells recruited to the sites of VEGF-induced neovascularization express both CD11b and NRP1 Immunofluorescence staining for CD11b (in red) and NRP1 (in green) on frozen sections from muscles 1 week after implantation of V Low-, V Med-, and V High-expressing myoblast clones or control cells (Ctrl). Essentially all CD11b-positive cells also expressed NRP1. Nuclei positive for NRP1, but not for CD11b, belong to endothelial cells. Scale bar = 25 μm.
Flow cytometry analysis confirmed that CD11b-positive monocytes isolated from the muscles implanted with VEGF-expressing cells were also NRP1-positive. The panels show representative data from two independent experiments.

**Figure EV3:**
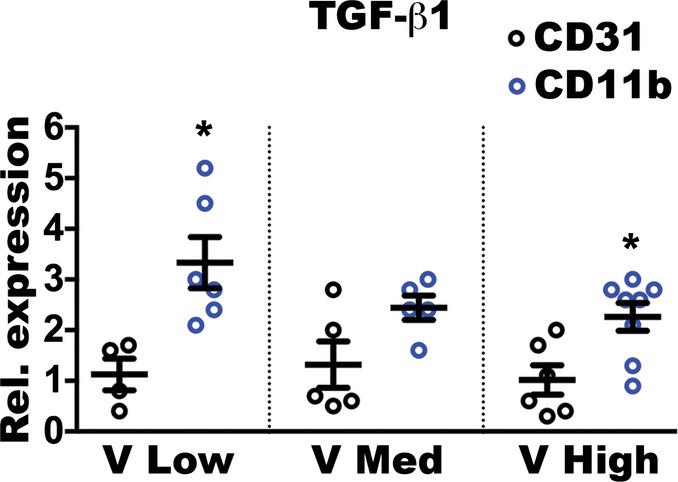
*Ex vivo* purified NEM express higher levels of TGF-β1 than endothelial cells TGF-β1 gene expression was quantified on NEM (CD11b) and endothelial cells (CD31) FACS-purified from muscles 1 week after implantation with V Low-, V Med-, and V High-expressing myoblast clones. The expression in CD11b-positive cells is shown as fold-change versus that in endothelial cells. Data represent individual values, with mean ± SEM (*n* = 4–7); *P < 0.05 by *t*-test with Welch’s correction: V Low— CD11b versus CD31 *P* = 0.0315; V High—CD11b versus CD31 *P* = 0.0281. Source data are available online for this figure.

Both VEGF and Sema3A are able to recruit NEM, which have been shown to promote smooth muscle cell recruitment and arteriogenesis through paracrine factor secretion (Zacchigna *et al*, 2008). NEM were previously shown to co-express NRP1 and the monocyte marker CD11b (Zacchigna *et al*, 2008). Both immunostaining and flow cytometry confirmed that essentially all CD11b^+^ cells recruited to muscles implanted with VEGF-expressing clones also expressed NRP1 (Fig EV2). Since NRP1 is also expressed on endothelium and pericytes, for clarity in subsequent experiments NEM were identified only by CD11b staining, as previously described (Zacchigna *et al*, 2008). One week after implantation of VEGF-expressing myoblasts, increased numbers of CD11b^+^ cells were recruited to the areas of active angiogenesis compared to controls. However, increasing VEGF levels impaired NEM recruitment in a dose-dependent fashion (Fig 3A), as confirmed by quantification of NEM frequency on histological sections in the different conditions (CD11b^+^ cells/cm of vessel length; Fig 3B). In order to analyze cell-specific changes in gene expression, CD31^+^ endothelial cells and CD11b^+^ NEM were isolated *ex vivo* by FACS from implanted muscles. Flow cytometry quantification confirmed the VEGF dose-dependent impairment in NEM recruitment (Fig 3C). As shown in Fig 3D, isolated endothelial cells downregulated *Sema3a* expression by 5-fold in a gradual and VEGF dose-dependent fashion, similar to the results obtained from whole-tissue analyses in Fig 2A, whereas neither *Pdgfb* nor *Tgfb1* expression was regulated by VEGF dose. Isolated CD11b^+^ cells expressed similar levels of both *Nrp1* and *Tgfb1* at all VEGF doses (Fig 3D), but expressed two- to three-fold more *Tgfb1* than endothelial cells, again regardless of VEGF dose (Fig EV3). Taken together, these results show that increasing VEGF doses specifically impaired endothelial expression of Sema3a and NEM recruitment, but did not regulate TGF-β1 expression by either endothelium or NEM, causing a reduction in total TGF-β1 indirectly through inhibition of NEM recruitment.

### Endogenous Sema3A is required for NEM recruitment

**Figure 4:**
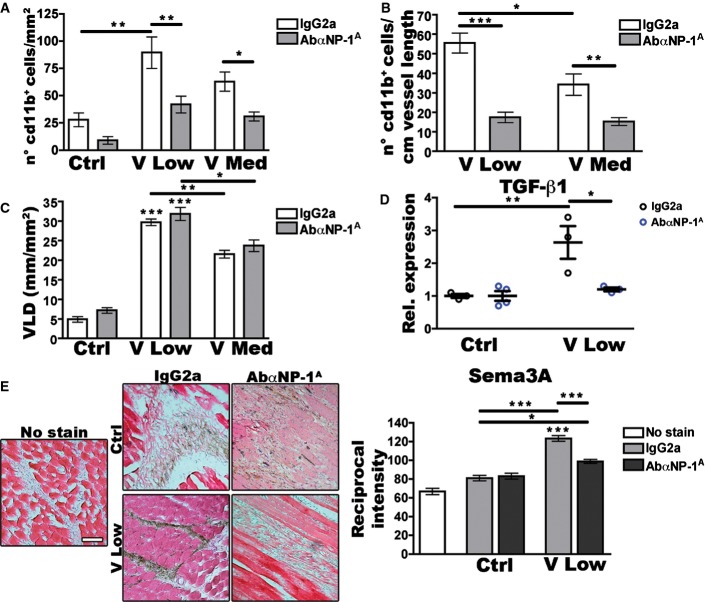
Sema3A/NRP1 binding is required for NEM recruitment, TGF-β1 upregulation, and Sema3A production Mice, implanted with control cells (Ctrl) or myoblasts expressing low (V Low) or medium (V Med) VEGF levels, were treated with the anti-NRP1^A^ antibody blocking Sema3A/NRP1 binding (AbαNRP1^A^) or with control IgG2a. Analyses were performed after 1 week. A, B Quantification of recruited NEM in sites of new angiogenesis by increasing VEGF doses, expressed as number of CD11b^+^ cells/mm^2^ of area (A) or /cm of vessel length (B). Data represent the mean ± SEM of individual images (*n*) acquired from three muscles/group. (A) Ctrl IgG2a, *n* = 11; Ctrl AbαNRP1^A^, *n* = 17; V Low IgG2a, *n* = 24; V Low AbαNRP1^A^, *n* = 28; V Med IgG2a, *n* = 25; V Med AbαNRP1^A^, *n* = 34; *P < 0.05, **P < 0.01 and ***P < 0.001 by Kruskal–Wallis analysis with Dunn’s multiple comparisons test: V Low IgG2a versus Ctrl IgG2a *P* = 0.0068; V Low IgG2a versus V Low AbαNRP1^A^ *P* = 0.0068; V Med IgG2a versus V Med AbαNRP1^A^ *P* = 0.0460. (B) V Low IgG2a versus V Low AbαNRP1^A^ *P* < 0.0001; V Low IgG2a versus V Med IgG2a *P* = 0.0193; V Med IgG2a versus V Med AbαNRP1^A^ *P* = 0.0046.
C Quantification of vessel length density generated by increasing VEGF doses after the different treatments. Data represent the mean ± SEM of individual images (*n*) acquired from three muscles/group: Ctrl IgG2a, *n* = 6; Ctrl AbαNRP1^A^, *n* = 6; V Low IgG2a, *n* = 19; V Low AbαNRP1^A^, *n* = 20; V Med IgG2a, *n* = 20; V Med AbαNRP1^A^, *n* = 25; *P < 0.05, **P < 0.01 and ***P < 0.001 by Kruskal–Wallis analysis with Dunn’s multiple comparisons test: V Low IgG2a versus Ctrl IgG2a *P* < 0.0001; V Low AbαNRP1^A^ versus Ctrl IgG2a *P* < 0.0001; V Low IgG2a versus V Med IgG2a *P* = 0.0014; V Low AbαNRP1^A^ versus V Med AbαNRP1^A^ *P* = 0.0179.
D *Tgfb1* gene expression in total skeletal muscles after the different treatments, expressed as fold-change versus the Ctrl IgG2a group. Data represent individual values, with mean ± SEM (*n* = 3 or 4, as indicated); *P < 0.05 and **P < 0.01 by one-way ANOVA with Bonferroni multiple comparisons test, after data normalization by logarithmic transformation; V Low IgG2a versus Ctrl IgG2a *P* = 0.0083; V Low IgG2a versus V Low AbαNRP1^A^ *P* = 0.0315.
E Immunohistochemistry for Sema3A protein on frozen muscle sections with H&E counterstaining. The intensity of Sema3A stain was quantified as reciprocal intensity on a scale out of 250. Scale bar = 100 μm. Data represent the mean ± SEM of individual images (*n*) acquired from three muscles/group: No stain, *n* = 3; Ctrl IgG2a, *n* = 23; Ctrl AbαNRP1^A^, *n* = 12; V Low IgG2a, *n* = 53; V Low AbαNRP1^A^, *n* = 38; *P < 0.05 and ***P < 0.001 by Kruskal–Wallis analysis with Dunn’s multiple comparisons test: V Low IgG2a versus No stain *P* = 0.0007; V Low IgG2a versus Ctrl IgG2a *P* < 0.0001; V Low AbαNRP1^A^ versus Ctrl IgG2a *P* = 0.0142; V Low IgG2a versus V Low AbαNRP1^A^ *P* = 0.0001. Source data are available online for this figure.

The NRP1 receptor on NEM can bind both Sema3A and VEGF (Zentilin *et al*, 2006; Zacchigna *et al*, 2008). To determine whether Sema3A expression was required for NEM recruitment, we performed loss-of-function experiments by systemic treatment with an antibody that recognizes the CUB domains (a1a2) of NRP1 and prevents its binding with Sema3A, but does not affect the interaction with VEGF (anti-NRP1^A^, YW64.3) (Liang *et al*, 2007; Pan *et al*, 2007). Specific inhibition of Sema3A/NRP1 interaction essentially abolished NEM recruitment in muscles injected with V Low and V Med myoblasts, reducing the CD11b^+^ cell number to control values (Fig 4A). The striking reduction was confirmed also after normalizing for the induced vessel length (Fig 4B). As expected, anti-NRP1^A^ did not affect the amount of angiogenesis induced by the different VEGF doses (Fig 4C). In agreement with the inhibition of locally recruited NEM, gene expression analysis on total muscle extracts showed that the upregulation of *Tgfb1* caused by low VEGF was abolished by inhibition of Sema3A/NRP1 binding (Fig 4D). Interestingly, the amount of endogenous Sema3A protein in low VEGF conditions, quantified by intensity of immunostaining on tissue sections, was also significantly reduced, though not completely abolished, by interference with Sema3A/NRP1 binding (Fig 4E). Taken together, these data suggest that Sema3A signaling through NRP1 is required for NEM recruitment and upregulation of TGF-β1 and, partly, also of Sema3A itself at sites of VEGF-induced angiogenesis.

### Increasing VEGF doses inhibit endothelial SMAD2/3 activation in newly induced vessels

**Figure 5:**
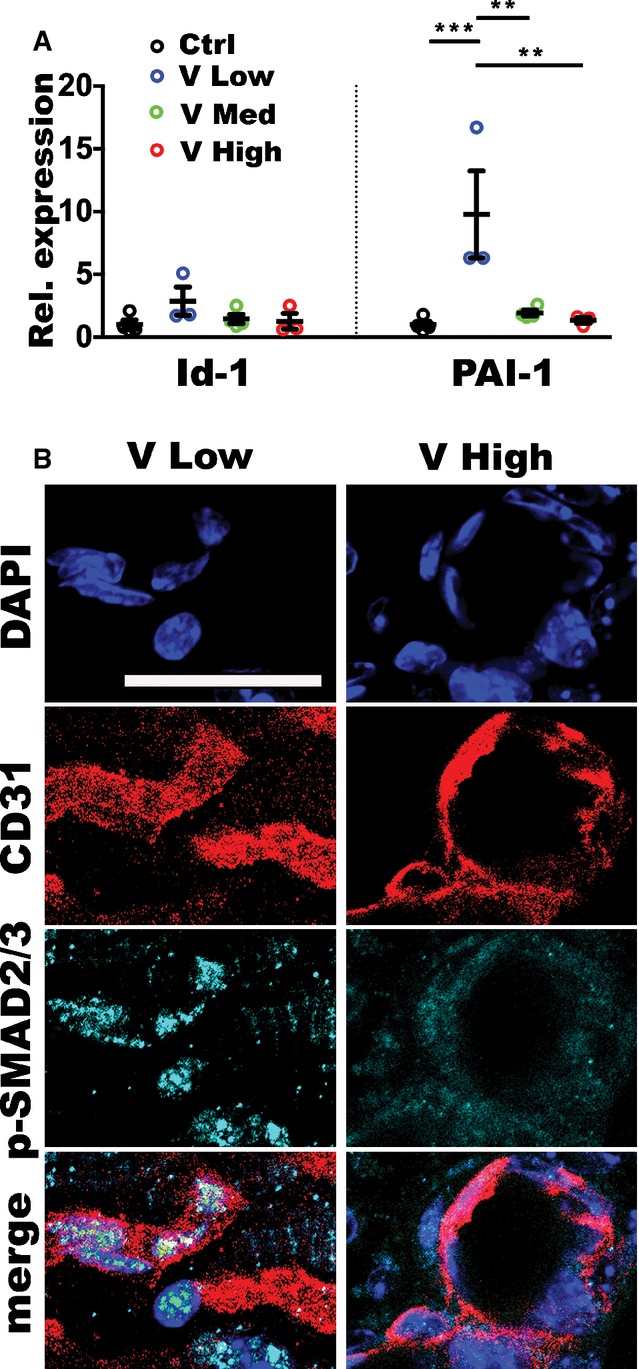
Increasing VEGF doses inhibit activation of endothelial SMAD2/3 Relative gene expression of *Id1*, specifically induced by activated SMAD1/5, and *Pai1*, specifically induced by activated SMAD2/3, in total muscle tissues 1 week after implantation with V Low-, V Med- and V High-expressing myoblast clones or control cells (Ctrl), expressed as fold-change versus the Ctrl group. Data represent individual values, with mean ± SEM (*n* = 3 or 4, as indicated); **P < 0.01 and ***P < 0.001 by one-way ANOVA with Bonferroni multiple comparisons test, after data normalization by logarithmic transformation; V Low versus Ctrl *P* = 0.0002; V Low versus V Med *P* = 0.005; V Low versus V High *P* = 0.0016.
Immunofluorescence staining of endothelium (CD31, in red), nuclei (DAPI, in blue), and phosphorylated SMAD2/3 (in cyan) on cryosections of similarly injected muscles confirmed that endothelial SMAD2/3 activation was inhibited by increasing VEGF doses. Scale bar = 10 μm; *n* = 3. Source data are available online for this figure.

TGF-β1 can promote both endothelial activation and quiescence by activating distinct intracellular signaling pathways through the phosphorylation of the SMAD1/5 or SMAD2/3 complexes, respectively (Goumans *et al*, 2002). Therefore, we determined whether TGF-β signaling was differentially activated in tissues exposed to increasing VEGF doses and which downstream pathway was preferentially stimulated. As shown in Fig 5A, the expression of Id-1, which is induced by the SMAD1/5 pathway and not by SMAD2/3 (Goumans *et al*, 2002), was not significantly increased in muscle tissue 1 week after implantation of the different VEGF-expressing clones compared to controls. Conversely, the expression of PAI-1, which is induced by the SMAD2/3 pathway and not by SMAD1/5 (Goumans *et al*, 2002), was robustly increased about 10-fold in tissues exposed to low VEGF levels compared to controls, but this upregulation was abolished by higher VEGF doses. Immunofluorescence staining confirmed that SMAD2/3 was phosphorylated and translocated into the endothelial nuclei of newly induced vessels 1 week after stimulation with low VEGF, but not with high VEGF (Fig 5B). Therefore, the VEGF dose-dependent downregulation of TGF-β1 expression specifically prevented the activation of the SMAD2/3 pathway, which mediates endothelial stabilization, in newly induced vascular structures.

### Endothelial Sema3A expression is directly inhibited by VEGF and induced by TGF-β1

**Figure 6:**
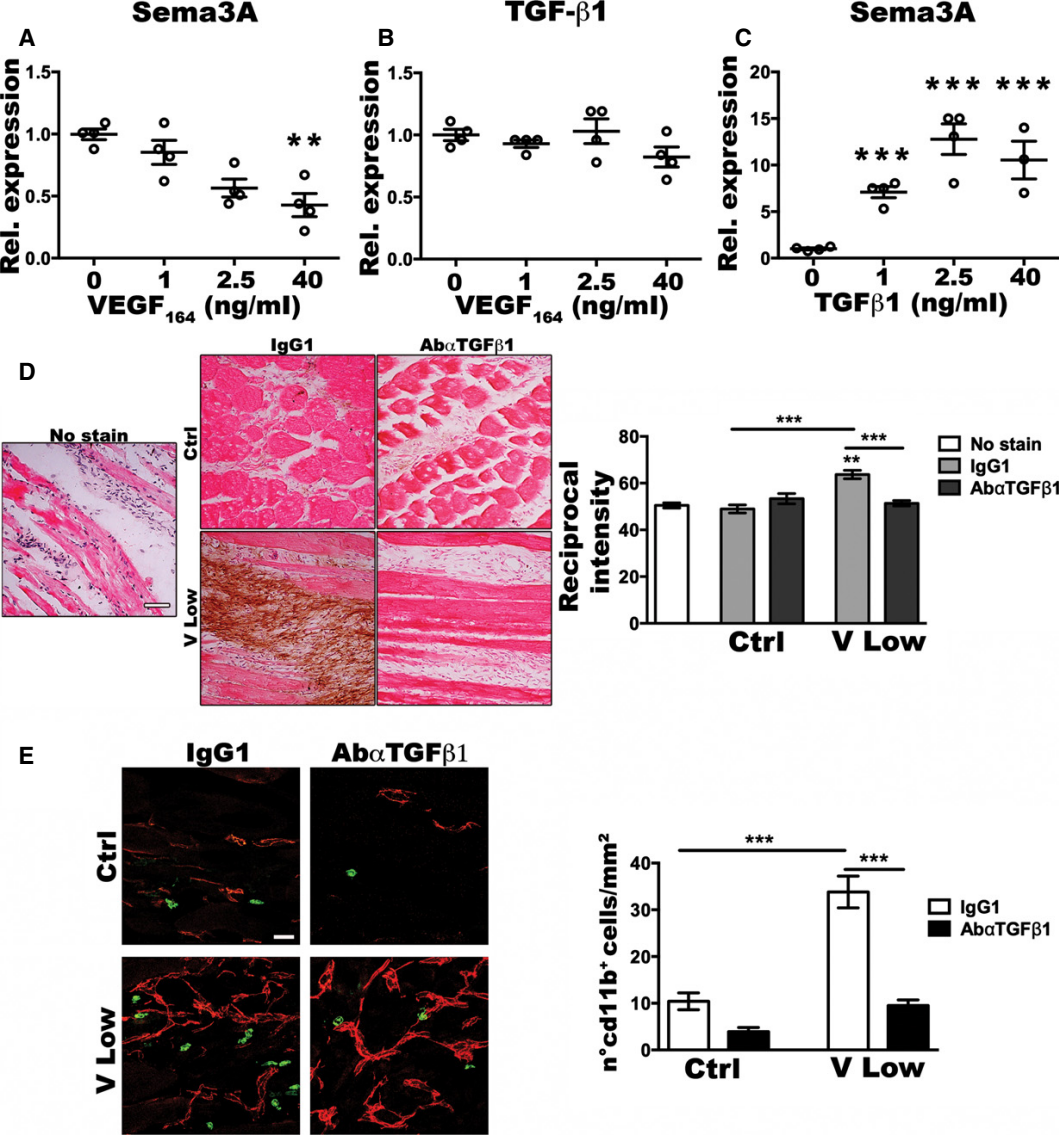
TGF-β1 regulates Sema3A production and NEM recruitment A–C Endothelial Sema3A expression is directly inhibited by VEGF and stimulated by TGF-β1 *in vitro*. Mouse aortic endothelial cells were stimulated with increasing doses of recombinant VEGF_164_ or TGF-β1 for 24 h. The expression of *Sema3a* and *Tgfb1* was quantified by qRT-PCR and expressed as fold-change versus the non-stimulated cells. VEGF dose dependently inhibited *Sema3a* expression (A), but did not affect that of *Tgfb1* (B), while TGF-β1 upregulated *Sema3a* expression in a dose-dependent fashion (C). Data represent individual values, with mean ± SEM (*n* = 4); **P < 0.01 and ***P < 0.001 by one-way ANOVA with Bonferroni multiple comparisons test, after data normalization by logarithmic transformation; Sema3A expression upon VEGF stimulation: 40 versus 0 *P* = 0.0041; TGF-β1 upon VEGF stimulation: no significant differences; Sema3A expression upon TGF-β1 stimulation: 1 versus 0 *P* < 0.0001; 2.5 versus 0 *P* < 0.0001; 40 versus 0 *P* < 0.0001.
D, E Inhibition of TGF-β1 abolishes Sema3A production and NEM recruitment *in vivo*. Mice, implanted with control cells (Ctrl) or myoblasts expressing low (V Low) VEGF levels, were treated with a TGF-β1 blocking antibody. Analyses were preformed after 1 week. (D) Immunohistochemistry for Sema3A protein on frozen muscle sections with H&E counterstaining. The intensity of Sema3A stain was quantified as reciprocal intensity on a scale out of 250. Scale bar = 50 μm. Data represent the mean ± SEM of individual images (*n*) acquired from three muscles/group: No stain, *n* = 10; Ctrl IgG1, *n* = 17; Ctrl AbαTGF-β1, *n* = 16; V Low IgG1, *n* = 23; V Low AbαTGF-β1, *n* = 28. **P < 0.01 and ***P < 0.001 by Kruskal–Wallis analysis with Dunn’s multiple comparisons test: V Low IgG1 versus No stain *P* = 0.0069; V Low IgG1 versus Ctrl IgG1 *P* < 0.0001; V Low AbαTGF-β1 versus V Low IgG1 *P* = 0.0002. (E) Quantification of recruited NEM (number of CD11b^+^ cells/mm^2^ of area). Scale bar = 25 μm. Data represent the mean ± SEM of individual images (*n*) acquired from three muscles/group: Ctrl IgG1, *n* = 20; Ctrl AbαTGF-β1, *n* = 19; V Low IgG1, *n* = 20; V Low AbαTGF-β1, *n* = 27; ***P < 0.001 by Kruskal–Wallis analysis with Dunn’s multiple comparisons test: V Low IgG1 versus Ctrl IgG1 *P* = 0.0005; V Low AbαTGF-β1 versus V Low IgG1 *P* < 0.0001. Source data are available online for this figure.

**Figure EV4:**
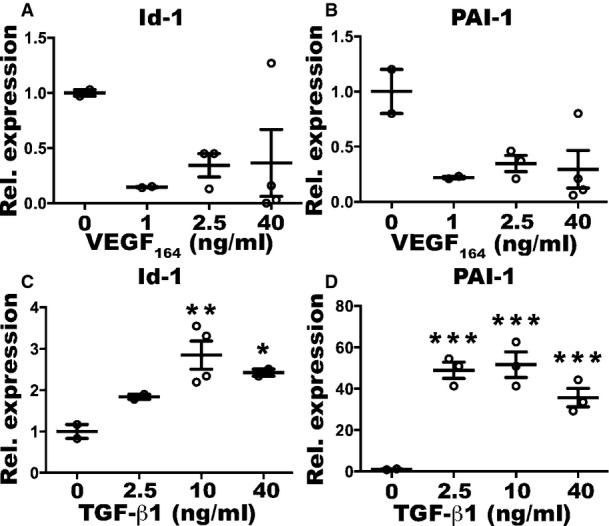
Expression of the TGF-β1 downstream genes Id-1 and PAI-1 is not stimulated by VEGF A–D Mouse aortic endothelial cells were stimulated *in vitro* with increasing doses of recombinant VEGF_164_ or TGF-β1 for 24 h. The expression of Id-1 and PAI-1 was quantified by qRT–PCR and expressed as fold-change versus that of non-stimulated cells. Increasing VEGF doses did not significantly change Id-1 (A) and PAI-1 expression (B), whereas TGF-β1 upregulated both (C, D). Data represent individual values, with mean ± SEM (*n* = 2–4); *P < 0.05, **P < 0.01, and ***P < 0.001 by one-way ANOVA with Bonferroni multiple comparisons test, after data normalization by logarithmic transformation; Id-1 and PAI-1 expression upon VEGF stimulation: no significant differences were detected; Id-1 expression upon TGF-β1 stimulation: 10 versus 0 *P* = 0.0058; 0 versus 40 *P* = 0.0247; PAI-1 expression upon TGF-β1 stimulation: all comparisons versus 0 *P* < 0.0001. Source data are available online for this figure.

In order to determine whether VEGF may regulate endothelial Sema3A expression directly or indirectly, mouse aortic endothelial cells were stimulated *in vitro* with increasing VEGF doses for 24 h before RNA extraction and gene expression analysis. Increasing VEGF doses directly downregulated endothelial expression of *Sema3a* (Fig 6A), but did not affect that of *Tgfb1* (Fig 6B), consistently with the results obtained in *ex vivo* isolated endothelial cells. Further, expression of the TGF-β1 downstream genes *Pai1* and *Id1* was not stimulated by any VEGF dose (Fig  EV4A and B), whereas both could be significantly upregulated by TGF-β1 treatment (Fig EV4C and D). However, TGF-β1 directly and dose-dependently upregulated endothelial *Sema3a* expression (Fig 6C). Therefore, both VEGF and TGF-β1 signaling regulate endothelial Sema3A expression, but with opposite effects.

These *in vitro* results raised the possibility that endothelial Sema3A expression during VEGF-induced angiogenesis may be actually induced by TGF-β1 rather than directly by VEGF. To determine whether TGF-β1 was required for Sema3A induction *in vivo*, we abrogated TGF-β1 signaling by systemic treatment with a specific blocking antibody (Wan *et al*, 2008). TGF-β1 blockade essentially abolished the upregulation of Sema3A production 1 week after implantation of V low myoblasts, reducing it to the same level as controls (Fig 6D). Consistently with the loss of Sema3A production, NEM recruitment by low VEGF was also abolished (Fig 6E). Therefore, TGF-β1 is required for the induction of Sema3A production by VEGF *in vivo*.

### Sema3A treatment promotes vascular stabilization without inhibiting angiogenesis

**Figure 7:**
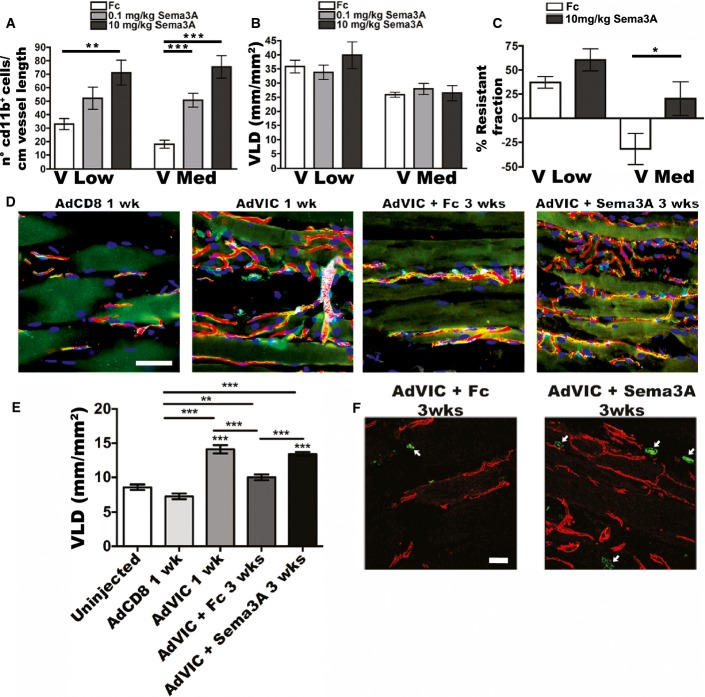
Sema3A restores NEM recruitment and accelerates vascular stabilization, without affecting the amount of induced angiogenesis A–C Mice, implanted with myoblasts expressing low (V Low) or medium (V Med) VEGF levels, were treated on days 4 and 6 after cell implantation with intramuscular injections of Sema3A-Fc (0.1 or 10 mg/kg of average muscle tissue weight), or with control Fc protein. (A) Quantification of the number of NEM/cm of vessel length in sites of new angiogenesis after 1 week. Data represent the mean ± SEM of individual images (*n*) acquired from 3 to 5 muscles/group: V Low Fc, *n* = 21; V Low 0.1 mg/kg, *n* = 22; V Low 10 mg/kg, *n* = 28; V Med Fc, *n* = 20; V Med 0.1 mg/kg, *n* = 30; V Med 10 mg/kg, *n* = 25; **P < 0.01 and ***P < 0.001 by Kruskal–Wallis analysis with Dunn’s multiple comparisons test: V Low Fc versus V Low 10 mg/kg *P* = 0.0027; V Med Fc versus V Med 0.1 mg/kg *P* = 0.001; V Med Fc versus V Med 10 mg/kg *P* < 0.0001. (B) Quantification of vessel length density (VLD) in treated muscles after 1 week. Data represent the mean ± SEM of individual images (*n*) acquired from 3 to 4 muscles/group: V Low Fc, *n* = 8; V Low 0.1 mg/kg, *n* = 20; V Low 10 mg/kg, *n* = 18; V Med Fc, *n* = 8; V Med 0.1 mg/kg, *n* = 23; and V Med 10 mg/kg, *n* = 27. Data were subjected to Kruskal–Wallis analysis with Dunn’s multiple comparisons test, and no significant differences were detected. (C) Vascular stabilization rate was determined 2 weeks after cell implantation by measuring vessel length density after abrogation of VEGF signaling by VEGF-Trap. Treatment with 10 mg/kg Sema3A-Fc did not affect the stabilization of angiogenesis induced by low VEGF, but significantly increased the resistant fraction of vessels induced by medium VEGF. Data represent the mean ± SEM of individual images (*n*) acquired from 3 to 4 muscles/group: V Low Fc, *n* = 32; V Low Sema3A, *n* = 14; V Med Fc, *n* = 28; V Med Sema3A, *n* = 33; *P < 0.05 by one-way ANOVA with Bonferroni multiple comparisons test: V Med Fc versus V Med Sema3A *P* = 0.0458.
D–F Mice received intramuscular injections of adenovirus expressing VEGF (AdVIC) or CD8 control (AdCD8) and were treated on days 4 and 6 after vector delivery with intramuscular injections of Sema3A-Fc (10 mg/kg of average muscle tissue weight) or control Fc protein. (D) Immunofluorescence staining of endothelium (CD31, in red), pericytes (NG2, in green), smooth muscle cells (α-SMA, in cyan), and nuclei (DAPI, in blue), showing vessel density and morphology 1 week after injection of adenoviral vectors alone (no treatment) or after 3 weeks and with Fc or Sema3A-Fc treatment. Scale bar = 50 μm. (E) Quantification of vessel length density (VLD) on the same samples shows that treatment with Sema3A-Fc accelerated stabilization of vessels induced by transient and uncontrolled VEGF expression. Data represent the mean ± SEM of individual images (*n*) acquired from 3 to 4 muscles/group: Uninjected muscles, *n* = 72; AdCD8 1 week, *n* = 68; AdVIC 1 week, *n* = 53; AdVIC+Fc 3 weeks, *n* = 78; AdVIC+Sema3AFc 3 weeks, *n* = 91; **P < 0.01 and ***P < 0.001 by Kruskal–Wallis analysis with Dunn’s multiple comparisons test: AdCD8 1 week versus AdVIC+Fc 3 weeks *P* = 0.0037; all other comparisons indicated *P* < 0.0001. (F) Immunofluorescence staining of endothelial cells (CD31, in red) and NEM (CD11b, in green) on frozen sections of muscles 3 weeks after injection of adenoviral vectors and with Fc or Sema3A-Fc treatment. White arrows indicate NEM. Scale bar = 50 μm. Source data are available online for this figure.

Based on these results, we asked whether gain of function of Sema3A signaling could reverse the impairment of vascular stabilization caused by increasing VEGF doses. Therefore, animals were treated with intramuscular injections of a recombinant Sema3A-Fc fusion protein at two doses (0.1 and 10 mg/kg of average muscle tissue weight) 4 and 6 days after implantation of V Low or V Med myoblasts in the same muscles. Seven days after myoblast injection, Sema3A-Fc caused a dose-dependent increase in CD11b^+^ cell recruitment at the sites of newly induced angiogenesis both in the presence of low and medium VEGF. Interestingly, already the lower dose (0.1 mg/kg) restored the NEM number in V Med muscles to a similar level as in the untreated V Low tissues, thereby abolishing the loss caused by the increasing VEGF dose (Fig 7A). Since Sema3A may also inhibit angiogenesis (Zacchigna *et al*, 2008; Maione *et al*, 2009), vessel length density was quantified in treated muscles, showing that Sema3A-Fc treatment at the doses used here did not affect the amount of vascular growth induced by either low or medium VEGF levels (Fig 7B).

To determine the functional effect of Sema3A delivery on vascular stabilization, VEGF signaling was abrogated by systemic treatment with VEGF-Trap and the resistant fraction of induced vessels was calculated as the percentage of vessel length density increase after Trap treatment compared to saline. Two weeks after myoblast implantation, mice treated with control Fc protein showed a vascular stabilization rate of 37 ± 6% in the presence of low VEGF, whereas vessels induced by medium VEGF doses completely regressed, confirming the results shown in Fig 1. However, local intramuscular treatment with 10 mg/kg of Sema3A-Fc caused the early stabilization of 20 ± 17% of vessels induced by medium VEGF doses, as well as an increase in the resistant fraction of angiogenesis induced by low VEGF to 60 ± 11% (Fig 7C). These results suggest that exogenous Sema3A can restore NEM recruitment and accelerate vascular stabilization, which are impaired by increasing VEGF doses.

Lastly, we sought to extend our findings, obtained with a controlled myoblast-based gene delivery platform, to a gene delivery system appropriate for clinical translation as a gene therapy approach. Therefore, we tested whether Sema3A treatment could accelerate stabilization of angiogenesis induced by intramuscular delivery of a VEGF-expressing adenoviral vector in immunocompetent C57/Bl6 mice. In this setting, the immune reaction against the adenoviral vector eliminates the delivered gene within 10 days (Dai *et al*, 1995), causing transient VEGF expression of insufficient duration to allow stabilization and persistence of newly induced vessels, similar to a clinical scenario with immunologically normal patients. Limb muscles of C57/Bl6 mice were injected with 1 × 10^8^ infectious units of an adenovirus expressing murine VEGF_164_ linked to a truncated form of CD8 as a cell-surface marker (AdVIC) or CD8 alone as a control (AdCD8). In the absence of treatment, 1 week after delivery, Ad-VEGF induced robust angiogenesis compared to the control vector and caused a 2-fold increase in vessel length density (Fig 7D and E), showing the initial efficacy of VEGF gene delivery. As expected, by 3 weeks, newly induced vessels had almost completely regressed in muscles treated with control Fc protein, yielding a VLD similar to uninjected control muscles that received no viral vector (Fig 7D and E). However, local treatment with two intramuscular doses of Sema3A-Fc on days 4 and 6 after adenoviral delivery caused complete stabilization of induced angiogenesis and its persistence by 3 weeks, maintaining VLD at the same level as after 1 week (Fig 7D and E). Further, treatment with Sema3A led to an increased NEM recruitment still after 3 weeks, compared to control Fc protein, even though treatment was stopped 2 weeks earlier (Fig 7F).

## Discussion

Here, we uncovered a novel function for Neuropilin-1-expressing monocytes to promote VEGF-independence of nascent blood vessels. Further, NEM recruitment during VEGF-induced angiogenesis requires Sema3A upregulation and inhibition of the endothelial Sema3A/NEM/TGF-β1 axis is a mechanism by which VEGF impairs vascular stabilization without affecting vascular maturation (pericyte recruitment). This impairment is VEGF dose-dependent and is caused by VEGF levels within the range that induces only normal and mature capillary networks and that is required for therapeutic benefit (Ozawa *et al*, 2004; von Degenfeld *et al*, 2006). Conversely, treatment with recombinant Sema3A counteracts the effects of increasing VEGF levels, restores NEM recruitment, and accelerates vascular stabilization, leading to persistent angiogenesis despite transient VEGF delivery.

**Figure 8:**
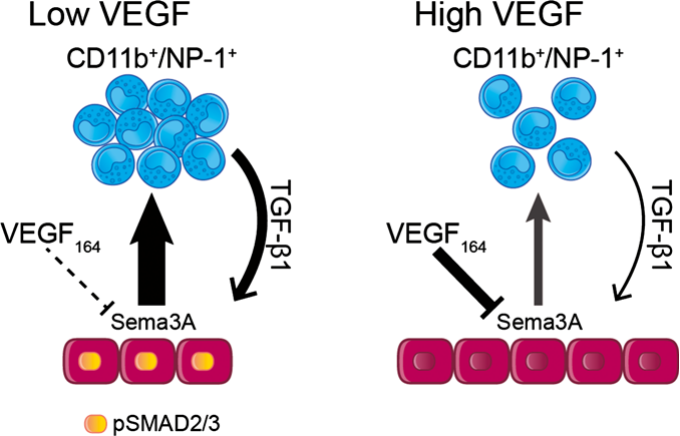
Working model for the VEGF dose-dependent regulation of vascular stabilization CD11b^+^/NP-1^+^ = CD11b- and Neuropilin1-expressing monocytes; pSMAD2/3 = phosphorylated SMAD2/3.

Taken together, our *in vivo* and *in vitro* results suggest a model for the VEGF dose-dependent regulation of vascular stabilization (Fig 8): (i) in the presence of low VEGF levels, activated endothelial cells robustly express Sema3A, which recruits large numbers of NEM that in turn lead to high levels of TGF-β1 in the tissue. TGF-β1 not only activates SMAD2/3 signaling in endothelial cells, known to induce endothelial quiescence and vessel stabilization, but also stimulates them to express further Sema3A, thereby providing a positive feedback loop to maintain the stabilizing signals. The function of this feedback mechanism is shown by the fact that a) blockade of Sema3A/NRP1 binding not only inhibited NEM recruitment and TGF-β1 expression, but also downregulated Sema3A itself (Fig 4), suggesting that NEM recruitment contributes to sustaining endothelial Sema3A production, and b) direct blockade of TGF-β1 abolished both Sema3A production and NEM recruitment despite VEGF expression at low levels (Fig 6D and E); (ii) on the other hand, higher levels of VEGF directly inhibit endothelial expression of Sema3A and lead to the reversal of these events, resulting in delayed stabilization of the newly induced vessels, despite pericyte coverage being unaffected.

Lack of pericyte coverage in the early stages of physiological angiogenesis (Benjamin *et al*, 1998) or in aberrant vascular structures induced by excessive VEGF (Dor *et al*, 2002; Ozawa *et al*, 2004) defines a plasticity window, whereby newly induced vessels are unstable and can regress. However, it is unclear whether pericytes are sufficient to prevent vessel regression. In fact, pericyte association showed a protecting role on vascular persistence in the retina under hyperoxic conditions and in tumors after VEGF withdrawal (Benjamin *et al*, 1998; Helfrich *et al*, 2010), but vessel regression after VEGF blockade has been described also for pericyte-covered vessels (Inai *et al*, 2004; von Tell *et al*, 2006). In agreement with the latter, we found that all normal capillaries induced by low and medium VEGF levels were similarly covered by pericytes by 2 weeks, but stabilization was still incomplete and further increased by 3 weeks, with the medium VEGF condition lagging behind low VEGF. Therefore, although pericyte recruitment is necessary, complete stabilization requires further steps that are independent of pericytes and can be impaired by increasing VEGF doses. Our *in vivo* and *in vitro* data suggest that the axis between endothelial Sema3A expression, NEM recruitment, and TGF-β1-induced phosphorylation of endothelial SMAD2/3 is such a mechanism.

On the other hand, aberrant structures induced by high VEGF always regressed after TRAP treatment, but they lacked pericytes and were coated by smooth muscle cells instead. Macro-vascular smooth muscle cells have distinct biological functions from capillary pericytes, providing mainly mechanical support rather than the complex molecular cross-talk of regulatory signals that pericytes exchange with endothelium (Jain, 2003). Further, although smooth muscle-coated normal arteries and veins are physiologically stable, the similarly smooth muscle-coated structures induced by high VEGF are functionally aberrant, as they grow progressively and behave as arterio-venous shunts (Zacchigna *et al*, 2007).

NEM have been described to produce paracrine factors capable of attracting smooth muscle cells *in vitro* (Carrer *et al*, 2012). However, it is unlikely that chemoattractant factors produced by NEM may be responsible for the observed switch in mural cell coverage between VEGF doses, as the condition where vascular structures acquire a smooth muscle coat (high VEGF) corresponds to the minimum amount of NEM recruitment (Fig 3). On the other hand, we have found before that, while low VEGF levels allow pericyte retention on the remodeling vessels, high VEGF levels cause pericytes to disappear in the early stages of vessel activation (Gianni-Barrera *et al*, 2013), consistently with the described anti-pericyte effect of VEGF through the formation of non-functional VEGF-R2/PDGF-Rβ heterodimers (Greenberg *et al*, 2008). The origin of smooth muscle cells that subsequently cover aberrant angioma-like structures induced in these conditions is currently unknown.

Blood flow and shear stress also can provide stabilizing signals to nascent vessels (Chen *et al*, 2012). However, available evidence suggests that these factors may not play a role in the observed impairment in vascular stabilization by VEGF dose. In fact, (i) the vascular networks induced by the different VEGF doses were similarly connected with the systemic circulation (Fig EV1); and (ii) we previously found that the amount of blood flow in vessels induced by the V Med myoblasts is actually higher than in those generated by low VEGF (von Degenfeld *et al*, 2006), despite showing here that stabilization is faster in the latter condition.

TGF-β receptor/SMAD signaling has been also involved in the process of endothelial to mesenchymal transition (EndMT): for example, spontaneous activation both of SMAD and non-SMAD TGF-β signaling in the endothelium of PAI-1-deficient animals led to EndMT and cardiac fibrosis (Ghosh *et al*, 2010; van Meeteren & ten Dijke, 2012). Interestingly, we found the highest upregulation of PAI-1 expression during angiogenesis induced by low VEGF, which caused the fastest stabilization rate with strong TGF-β1 expression and SMAD2/3 activation. Further, we could not detect any endothelial cell co-expressing α-SMA and CD31, suggesting that TGF-β1 expression did not lead to EndMT during low VEGF-induced angiogenesis. In fact, the pleiotropic effects of TGF-β1 signaling depend on the specific set of activated downstream transcription factors and selective SMAD2/3 activation, which we found in low VEGF conditions, specifically promotes vessel stabilization by inhibiting endothelial migration and proliferation that are instead required for EndMT (Goumans *et al*, 2002; van Meeteren & ten Dijke, 2012). Indeed, it has been shown that TGF-β1 stimulation of endothelial proliferation or quiescence through Alk1 and Alk5, respectively, depends on its dose: in fact, Alk1 is preferentially activated by low TGF-β1, whereas at higher doses Alk5 becomes prevalent (Goumans *et al*, 2002), consistently with our findings, where the most rapid stabilization is caused by the highest TGF-β1 upregulation. Further, PAI-1, which is induced by TGF-β1 activation of the Alk5 and SMAD2/3 pathway, contributes to vessel stabilization by preventing degradation of the provisional matrix deposited around new vessels and favoring the establishment of new basal lamina (Potente *et al*, 2011), again consistently with our finding that PAI-1 was specifically and robustly induced in the low VEGF conditions, leading to the fastest stabilization.

To our knowledge, these results provide the first evidence that NEM recruitment to sites of VEGF-induced angiogenesis requires Sema3A and that endothelial production of Sema3A is regulated, with opposing effects, by VEGF and TGF-β1. It remains to be completely elucidated by which mechanism Sema3A is initially upregulated upon endothelial activation. However, our *in vitro* and *in vivo* data suggest that this is not a direct effect of VEGF itself, and strongly implicate a role for TGF-β1. In fact, *in vitro* VEGF treatment did not increase basal Sema3A expression of cultured EC, whereas TGF-β1 could do so robustly (Fig 6), and *in vivo* treatment with a blocking antibody showed that TGF-β1 is required for Sema3A expression upon VEGF delivery. Interestingly, recent data in a model of liver regeneration show that endothelial expression of TGF-β1 is stimulated by autocrine Ang-2 signaling (Hu *et al*, 2014). These findings, together with our data on the role of TGF-β1, suggest an explanation for the dual behavior of Sema3A expression at different VEGF doses. In fact, upon angiogenic activation by VEGF, endothelial cells release Ang-2 (Augustin *et al*, 2009), but we found here that endothelial Ang-2 expression does not increase with higher VEGF doses (Fig 2A). Therefore, it is possible to speculate that, in low VEGF conditions, initial Ang-2 release upregulates endothelial TGF-β1, which in turn stimulates Sema3A expression and initiates the NEM/TGF-β1 positive feedback loop described above, leading to sustained Sema3A production by NEM-produced TGF-β1. However, as VEGF dose increases, Ang-2 expression is not changed (Fig 2A) and the direct inhibition of Sema3A expression by VEGF may become prevalent, leading to the reversal of the NEM/TGF-β1 axis. Testing of this hypothesis will require *ad hoc* experiments with pharmacologic blockade or genetic deficiency of Ang-2.

The direct effects of Sema3A on endothelium have been also shown to inhibit VEGF-induced angiogenesis (Zacchigna *et al*, 2008; Maione *et al*, 2009), both through competition for the shared receptor NRP1 (Miao *et al*, 1999) and by inducing endothelial production of a decoy VEGF receptor, soluble Flt-1 (Zygmunt *et al*, 2011). However, we did not observe any such effect. In fact, 2 weeks after cell injection, low VEGF, which is the condition with the highest amount of Sema3A expression, induced a greater amount of vascular growth than both medium and high doses, which instead caused significant Sema3A downregulation (Fig 1B). This apparent discrepancy may reflect a key role for the ratio between VEGF and Sema3A doses. In fact, when overexpressed at much greater than physiological levels through adeno-associated virus (AAV) vectors, Sema3A has been found to inhibit angiogenesis both by similarly overexpressed VEGF in AAV-treated muscles (Zacchigna *et al*, 2008) and by lower endogenous VEGF levels in tumor models (Maione *et al*, 2009, 2012). On the other hand, also endogenous levels of Sema3A have been shown to restrain angiogenesis induced by endogenous VEGF levels in tumors (Maione *et al*, 2009). However, in our work, VEGF was overexpressed through constitutive retroviral vectors in transduced myoblast populations, which were previously shown to generate tissue levels of VEGF several fold higher than the maximum induced by ischemia (von Degenfeld *et al*, 2006), whereas Sema3A was not overexpressed and was regulated from the endogenous promoter. In agreement with our data, previous results obtained with different ratios of Sema3A- and VEGF-expressing AAV showed that the anti-angiogenic effect was lost at a Sema3A:VEGF vector ratio of 1:100, whereas NEM recruitment was still effectively increased (Zacchigna *et al*, 2008). Taken together, these results suggest that the anti-angiogenic effect of Sema3A depends on its relative dose to VEGF and can be overcome by a sufficient amount of VEGF, while the NEM-recruiting function of Sema3A is active even at very low doses.

In conclusion, our results show that increasing VEGF doses impair vessel stabilization by directly inhibiting the Sema3A/NEM/TGF-β1 axis, while treatment with recombinant Sema3A-Fc restores NEM recruitment and accelerates vascular stabilization, without inhibiting vascular growth and despite transient VEGF expression by clinically relevant adenoviral vectors. This finding has implications for the design of safe and effective approaches for therapeutic angiogenesis. In fact, we have previously found that VEGF doses within the range inducing only normal angiogenesis are not therapeutically equivalent (von Degenfeld *et al*, 2006). In particular, the lower doses, which we found here to allow the fastest stabilization, are not effective to restore blood flow in ischemia, whereas functional improvement requires higher doses, which induce similarly normal vessels, but of larger size, and promote effective collateral arteriogenesis (von Degenfeld *et al*, 2006), but also inhibit Sema3A expression and delay stabilization. Furthermore, prolonged VEGF expression raises safety concerns, but, while transient delivery is desirable, it is insufficient to achieve effective stabilization and persistence of the induced vessels. However, based on our findings, co-delivery of Sema3A represents a promising target to accelerate stabilization of micro-vascular networks induced by therapeutic doses of VEGF, thereby enabling short-term and safer therapeutic approaches without compromising efficacy.

## Materials and Methods

### Cell culture

Primary myoblasts isolated from C57BL/6 mice, already transduced to express the β-galactosidase marker gene (lacZ), were further infected at high efficiency (Springer & Blau, 1997) with retroviruses carrying the cDNA of murine VEGF_164_ linked through an Internal Ribosome Entry Sequence (IRES) to a truncated murine CD8a as a FACS-sortable marker (Misteli *et al*, 2010). Early-passage myoblast clones were isolated using a FACS Vantage SE cell sorter (Becton–Dickinson, Basel, Switzerland) as described (Misteli *et al*, 2010). All myoblast populations were cultured in 5% CO_2_ on collagen-coated dishes, with a growth medium consisting of 40% F10, 40% DMEM (Sigma-Aldrich Chemie GmbH, Steinheim, Germany), and 20% fetal bovine serum (HyClone, Logan, UT), supplemented with 2.5 ng/ml FGF-2 (R&D Systems, Abingdon, UK), as described (Banfi *et al*, 2002).

### VEGF_164_ ELISA measurements

The production of VEGF_164_ in cell culture supernatants was quantified using a Quantikine mouse VEGF Immunoassay ELISA kit (R&D Systems Europe, Abingdon, UK). 1 ml of medium was harvested from myoblasts in one 60-mm dish, following a 4-h incubation, filtered, and analyzed in duplicate. Results were normalized by the number of cells in the dish and time of exposure to medium. Four dishes of cells were assayed per cell type (*n* = 4).

### *In vivo* implantation of myoblasts

Six- to twelve-week-old immunodeficient SCID CB17 mice (Charles River Laboratories, Sulzfeld, Germany) were used to avoid any immunologic response to myoblasts expressing xenogenic LacZ protein. Myoblasts were dissociated in trypsin and resuspended at a concentration of 10^8^ cells/ml in sterile PBS with 0.5% BSA (Sigma-Aldrich Chemie GmbH, Steinheim, Germany), and 10^6^ myoblasts were injected into the *Tibialis anterior* and *Gastrocnemius* muscles in the lower hind limb, using a syringe with a 29.5-gauge needle, as previously described (Ozawa *et al*, 2004). All experiments were performed with similar number of samples from both muscle locations, and the results were pooled together.

### Recombinant adenovirus production and *in vivo* implantation

Recombinant adenoviruses expressing mouse VEGF_164_ were produced using the Adeno-X™ Expression System (Clontech, Saint-Germain-en-Laye, France) according to the manufacturer’s recommendations. Constructs also expressed a truncated version of CD8 as a cell surface marker, as previously described (Banfi *et al*, 2012). Briefly, target genes were cloned into the pShuttle vector, sub-cloned into the Adeno-X viral DNA, and used to transfect HEK293 cells with Fugene HD reagent (Roche Applied Science, Basel, Switzerland). After 1 week, viral particles were collected from transfected cells by repeated freezing–thawing and used for re-infection of fresh HEK293. After 4–5 lysis and infection cycles, viral particles were collected and purified by a double cesium chloride gradient. Viral titer was determined as infectious units after serial infection of HEK293 cells at different multiplicities of infection, as previously described (Gueret *et al*, 2002). Adenoviral vectors were diluted in physiological solution and injected in *Tibialis anterior* and *Gastrocnemius* muscles in the lower hind limb of immunocompetent C57/Bl6 mice at the titer of 1 × 10^8^ infectious units/injection, with a 29.5-gauge needle syringe.

### VEGF-Trap_R1R2_ treatment

Mice were treated with 25 mg/kg of VEGF-Trap_R1R2_ (Aflibercept, Regeneron Pharmaceuticals Inc., Tarrytown, NY, USA) in 100 μl of PBS or with vehicle (100 μl of PBS alone) intraperitoneally (i.p.) 2 and 4 days before tissue harvesting, as described (Ozawa *et al*, 2004).

### Anti-NRP1^A^ antibody treatment

Animals were treated systemically by i.p. injection of the blocking antibody anti-NRP1^A^ (YW64.3, Genentech Inc., South San Francisco, CA, USA) (Liang *et al*, 2007) in PBS with 0.5% BSA (10 mg/kg) at the time of myoblast implantation (day 0) and after 3 and 6 days, according to the previously published treatment schedule (Pan *et al*, 2007). IgG2a antibody (10 mg/kg in PBS with 0.5% BSA; Lubio Science, Lucerne, Switzerland) was given i.p. as control.

### Anti-TGF-β1 antibody treatment

Animals were treated systemically by i.p. injection of the specific blocking antibody anti-TGF-β1 (Clone 9016, R&D Systems) in PBS with 0.5% BSA (100 μg/animal/dose) at the time of myoblast implantation (day 0) and after 2, 4, and 6 days, according to the previously published treatment schedule (Wan *et al*, 2008). IgG1 antibody in PBS with 0.5% BSA (100 μg/animal/dose; R&D Systems) was given i.p. as control.

### Sema3A-Fc treatment

*Tibialis anterior* and *Gastrocnemius* muscles were injected 4 and 6 days after injection of myoblasts or adenoviral vectors with Sema3A-Fc (R&D Systems) at a dose of either 0.1 or 10 mg/kg of the average muscle weight (60 mg for *Tibialis* and 120 mg for *Gastrocnemius*, corresponding to either 6 or 60 μg and 12 or 120 μg of Sema3A-Fc per single injection, respectively) or equivalent amounts of Fc fragment (Abcam, Cambridge, UK) in 15 μl of sterile PBS with 0.5% BSA.

### Tissue staining

Mice were anesthetized with ketamine (100 mg/kg) and xylazine (10 mg/kg) and sacrificed by intravascular perfusion with 1% paraformaldehyde in PBS pH 7.4. *Tibialis anterior* and *Gastrocnemius* muscles were harvested, post-fixed in 0.5% paraformaldehyde in PBS for 2 h, cryoprotected in 30% sucrose in PBS overnight at 4°C, embedded in OCT compound (CellPath, Newtown, Powys, UK), frozen in freezing isopentane, and cryosectioned. As areas of effect were limited to the small portions of muscle corresponding to the implantation sites, all analyses were performed on images taken from the areas of engraftment, which were unequivocally identified by tracking implanted myoblasts by X-gal staining (20-μm sections) or adenoviral infection sites by the typical mononuclear infiltrate with H&E (10-μm sections) in adjacent serial sections, as described previously (Ozawa *et al*, 2004). Sections of 10 μm in thickness were stained with the following primary antibodies and dilutions: rat monoclonal anti-mouse CD31 (clone MEC 13.3, BD Biosciences, Basel, Switzerland) at 1:100 or hamster monoclonal anti-mouse CD31 (clone 2H8, Millipore, Merck, Germany) at 1:200; mouse monoclonal anti-mouse α-SMA (clone 1A4, MP Biomedicals, Basel, Switzerland) at 1:400; rabbit polyclonal anti-NG2 (Millipore) at 1:200; rat monoclonal anti-CD11b (clone M1/70, Abcam, Cambridge, UK) at 1:100; rabbit polyclonal anti-NRP1 (Abcam) at 1:50; rabbit polyclonal anti-p-SMAD2/3 (Santa Cruz Biotechnology, Santa Cruz, CA, USA) at 1:100; and rabbit polyclonal anti-Sema3A (Abcam) at 1:50. Fluorescently labeled secondary antibodies (Invitrogen, Basel, Switzerland) were used at 1:200. The Sema3A primary antibody was detected with a biotinylated or with a peroxidase-labeled anti-rabbit secondary antibody. The first chromogenic signal was developed with Vectastain ABC kits (Vector Laboratories) and the Fast Red kit (Dako, Baar, Switzerland), and the second with 3,3′-diaminobenzidinetetrahydrochloride (Sigma).

To study vessel perfusion, 100 μg of fluorescein isothiocyanate (FITC)-labeled *Lycopersicon esculentum* lectin in 50 μl (Vector Laboratories) was injected into the femoral vein and allowed to circulate for 4 min before intravascular perfusion with 1% paraformaldehyde and leg muscle collection as described above.

### RNA *in situ* hybridization (ISH)

Expression of Sema3A and CD31 mRNA was visualized in individual cells in tissue sections with a highly sensitive and specific ISH system, according to the manufacturer’s instructions (QuantiGene ViewRNA, Affymetrix UK, High Wycombe, UK). Briefly, OCT-embedded muscles were cryosectioned (10 μm thickness), mounted onto Superfrost Plus Gold glass slides (Thermo Fischer Scientific, Wohlen, Switzerland), and kept at −80°C until use. Slides were fixed with 4% formaldehyde for 16–18 h at 4°C, washed, dehydrated in ethanol, and pretreated by boiling for 1 min in pretreatment solution, followed by 10-min digestion in Protease QF (both from Affymetrix). Sections were hybridized for 2 h at 40°C with QuantiGene ViewRNA probes specific for the mouse Sema3A and mouse CD31 RNA sequences (VB1-11132-06 and VB6-12921-01, respectively, Affymetrix). For each experiment, one section was hybridized with probe diluent only (Probe Set Diluent QT, Affymetrix) as negative control. Label Probe oligonucleotides, conjugated to alkaline phosphatase (LP-AP) type 1 or type 6, were added, followed by appropriate substrates: an LP-AP type 6 probe was used with Fast Blue substrate for Sema3A detection, followed by LP-AP type 1 probe with Fast Red substrate for CD31 detection. Finally, slides were counterstained with Meyer’s hematoxylin and DAPI and mounted with aqueous mounting medium (Dako Ultramount Permanent Mounting Media S1964).

### ISH image acquisition and quantification

The images were acquired with a laser scanning confocal microscope (LSM710, Carl Zeiss Microscopy, Göttingen, Germany) and Zen2 software (Carl Zeiss Microscopy). For each muscle section, the area of injection was selected and high-power images (212.3 × 212.3 μm) were acquired with a 40× objective. Pseudo-colors were assigned to each fluorescent dye during acquisition (red for Fast Red, green for Fast Blue and blue for DAPI). Color channels of the acquired images were separated and exported as TIF files. Nuclei were identified by DAPI staining. Red and green dots, corresponding to the staining for CD31 and Sema3A mRNA, respectively, were identified and all cells positive for either or both were counted manually in each field.

### Histological analysis

Vessel length density (VLD) was quantified in fluorescently immunostained cryosections as described (Ozawa *et al*, 2004). Briefly, 10-15 fields per muscle (*n* = 4 muscles/group) were analyzed by tracing the total length of vessels in the acquired field and dividing it by the area of the fields. Vessel stabilization was calculated as the fraction of newly induced vessels, defined as the amount of VLD in excess of the pre-existing VLD measured on control uninjected muscles (VLD-ctrl), that survived after VEGF-Trap treatment, according to the formula (VLD-Trap – VLD-ctrl)/(VLD-vehicle – VLD-ctrl), where VLD-Trap = VLD after treatment with VEGF-Trap and VLD-vehicle = VLD after treatment with vehicle alone. All images were acquired using an Olympus BX61 microscope (Olympus, Volketswil, Switzerland) and analyzed with Cell Sense software (Olympus, Volketswil, Switzerland).

The quantification of vessel perfusion was performed on sections of leg muscles harvested after intravascular staining with fluorescent lectin, as described above. After co-staining with a fluorescent anti-CD31 antibody, the total lengths of lectin-positive and CD31-positive vascular structures in each field were traced and the vessel perfusion index was calculated as the ratio between the two values.

The quantification of pericyte coverage was performed on sections of leg muscles after immunostaining for endothelium (CD31) and pericytes (NG2) as previously described (Li *et al*, 2011). Briefly, the CD31- and NG2-positive areas were measured by ImageJ software (http://rsb.info.nih.gov/ij/), and the pericyte coverage index was calculated as the ratio between the two values (Li *et al*, 2011).

The quantification of CD11b^+^ cells was performed on sections of leg muscles after immunostaining for endothelium (CD31) and CD11b, and normalizing the absolute number of CD11b^+^ cells either by vessel length or tissue area.

The immunohistochemistry staining for Sema3A was quantified as reciprocal intensity by the standard intensity function in the open source Fiji software (ImageJ) (http://fiji.sc/Fiji) as described (Nguyen *et al*, 2013). Briefly, since the maximum intensity value of an RGB image analyzed in ImageJ is 250, the intensity of a stained region of interest was subtracted from 250, thereby deriving a reciprocal intensity that is directly proportional to the amount of chromogen.

For vessel perfusion, pericyte coverage, CD11b^+^ cell frequency, and Sema3A quantifications, 3–5 fields were analyzed in each muscle (*n* = 4 muscles/group). All images were taken with a Carl Zeiss LSM710 3-laser scanning confocal microscope (Carl Zeiss, Feldbach, Switzerland) or Olympus BX61 microscope, and analyses were conducted with Cell Sense software (Olympus).

No blinding was performed, but analyses were performed by two independent investigators.

### *Ex vivo* cell isolation by FACS

Pools of 20 limb muscles of SCID CB17 mice (*Tibialis anterior* and *Gastrocnemius*) were harvested 7 days after myoblast injection and processed as a single sample (*n* = 4–7 samples/group). Tissues were minced, digested with DNase (0.2 mg/ml, Sigma-Aldrich), Collagenase type IV (484 U/ml, Worthington), Collagenase I (0.5 mg/ml; Sigma-Aldrich), and Collagenase II (0.5 mg/ml, Sigma-Aldrich) in low-glucose DMEM (Sigma-Aldrich) for 45 min at 37°C under constant shaking, filtered through 100- and 70-μm cell strainers (BD Biosciences), and washed twice in PBS. Cells were stained at 4°C for 30 min with the following fluorescently labeled antibodies: PE-anti-mouse CD31 (clone 390; BioLegend, San Diego, CA, USA) at 1:200; PE-Cy7-anti-mouse CD11b (clone M1/70, BioLegend) at 1:1,000. CD31^+^ and CD11b^+^ cells were isolated with an Influx cell sorter (BD Biosciences). RNA was extracted, and gene expression analysis was performed as described below.

**The paper explained**

**Problem**

Vascular endothelial growth factor (VEGF) is the master regulator of angiogenesis and its delivery is an attractive approach to grow new blood vessels to treat ischemic diseases, an approach named therapeutic angiogenesis. A current challenge is that, while prolonged expression is required to achieve persistence of newly induced vessels and provide lasting therapeutic benefit, a short duration of treatment is desirable to ensure safety, as sustained expression can cause dysfunctional angiogenesis and the growth of angioma-like vascular tumors. Therefore, there is a need to identify molecular targets to accelerate vascular stabilization despite short-term VEGF delivery. To address this need, here we investigated whether VEGF dose regulates vascular stabilization and the underlying mechanism.

**Results**

Specific VEGF doses, in a therapeutically relevant range, were delivered to the clinical target tissue of skeletal muscle of mice, and VEGF signaling was abrogated after 10 and 17 days by treatment with Aflibercept, a receptor-body clinically approved for anti-VEGF therapy in ophthalmology and oncology indications. Increasing VEGF doses impaired vessel stabilization without affecting either pericyte coverage or functional perfusion, but rather through a novel mechanism involving direct inhibition of the expression of the Neuropilin ligand Semaphorin3A (Sema3A) by endothelium, leading to impaired recruitment of Neuropilin-expressing monocytes (NEM) and reduced tissue levels of transforming growth factor-β1 (TGF-β1). Interestingly, NEM recruitment was amplified and maintained by a positive feedback loop, whereby TGF-β1, secreted by Sema3A-recruited NEM, was required to induce and maintain production of Sema3A by endothelium. Blocking experiments showed that Sema3A signaling through Neuropilin-1 was required to start the NEM/TGF-β1 axis. Conversely, treatment with recombinant Sema3A rescued both NEM recruitment and vascular stabilization impaired by increasing VEGF doses, and enabled vessel persistence despite transient VEGF expression by clinically relevant adenoviral vectors.

**Impact**

These findings suggest a strategy to achieve persistent new vasculature despite limited duration of VEGF expression through co-delivery of Sema3A and stimulation of NEM recruitment, thereby improving the safety of therapeutic angiogenesis.

### *In vitro* endothelial cell assays

Mouse aortic endothelial cells (MAEC) were isolated as described (Kobayashi *et al*, 2005) and cultured in high-glucose DMEM (Sigma-Aldrich) supplemented with 10% FBS (HyClone, Logan, UT), 1 mM sodium pyruvate, 0.1 mM MEM Non-Essential Amino Acids, 2 mM glutamine, 100 U/ml penicillin, and 100 μg/ml streptomycin (Life Technologies, Zug, Switzerland). 1 × 10^5^ cells/well were seeded into 24-well plates, cultured to confluence, and then stimulated with recombinant mouse VEGF-A_164_ or human TGF-β1 (R&D Systems, Abingdon, UK) at different concentrations (0, 1, 2.5, and 40 ng/ml) in DMEM with 0.5% FBS at 37°C. After 24 h of stimulation, cells were collected for RNA extraction (*n* = 4 samples/group).

### Quantitative real-time PCR

For RNA extraction from total muscles, freshly harvested tissue was disrupted using a Qiagen Tissue Lyser (Qiagen, Basel, Switzerland) in 1 ml TRIzol Reagent for 100 mg of tissue (Life Technologies). Total RNA was isolated from lysed tissues, *in vitro* cultured MAEC or *ex vivo* FACS-sorted cells with an RNeasy Mini Kit (Qiagen) according to the manufacturer’s instruction.

RNA from total muscles and MAEC was reverse-transcribed into cDNA with the Omniscript Reverse Transcription kit (Qiagen) at 37°C for 60 min, whereas RNA from *ex vivo* sorted cells was reverse-transcribed into cDNA with the Sensiscript RT kit (Qiagen). Quantitative Real-Time PCR (qRT-PCR) was performed on an ABI 7300 Real-Time PCR system (Applied Biosystems). Expression of genes of interest was determined using the following TaqMan gene expression assays (Life Technologies): *Pdgfb* (Mm00440677_m1); *Tgfb1* (Mm01178820_m1); *Ang1* (Mm0045603_m1); *Ang2* (Mm00545822_m1); *Sema3a* (Mm00436469_m1); *Id1* (Mm00775963_g1); and *Pai1* (Mm00435860_m1). Reactions were performed in triplicate for each template, averaged, and normalized to expression of the *Gapdh* housekeeping gene (Mm03302249_g1).

qRT–PCR was also performed on total genomic DNA to quantify the number of myoblasts engrafted after implantation by measuring the amount of stably integrated LacZ retroviral construct. A reference curve was constructed for each of the different myoblast clonal populations with a 10-fold dilution series between 1 and 10^6^ cells, whereby the Δ*C*_t_ data of each sample could be transformed into the corresponding absolute cell numbers. LacZ primer and probe sequences and reaction concentrations were previously published (Mujagic *et al*, 2013).

### Statistics

Data are presented as mean ± standard error. The significance of differences was assessed with the GraphPad Prism 6 software (GraphPad Software). The normal distribution of all data sets was tested, and, depending on the results, multiple comparisons were performed with the parametric one-way analysis of variance (ANOVA) followed by the Bonferroni test, or with the nonparametric Kruskal–Wallis test followed by Dunn’s post-test. Gene expression data representing fold-changes versus control, which are asymmetrically distributed, were first normalized by logarithmic ln(y) transformation and then analyzed by one-way ANOVA followed by the Bonferroni test for multiple comparisons, or by *t*-test with Welch’s correction for single comparisons; *P* < 0.05 was considered statistically significant.

### Animal studies

All animal studies were performed in accordance with the Swiss Federal guidelines for animal welfare, after approval from the Veterinary Office of the Canton of Basel-Stadt (Basel, Switzerland; Permit 2071). Mice of 8–12 weeks of age, with equal representation of both genders, were randomly assigned to experimental groups, with a minimum of *n* = 4 mice/group.

## Supporting Information

Appendix

Expanded View Figures PDF

Review Process File

Source Data for Figure 7

Source Data for Expanded View and Appendix
