# Supplementary material for: VEGF dose regulates vascular stabilization through Semaphorin3A and the Neuropilin-1+ monocyte/TGF-β1 paracrine axis
Source: EMBO Mol Med. 2015 Sep 7;7(10):1366–84. doi: 10.15252/emmm.201405003 (PMC4604689; doi:10.15252/emmm.201405003)
Supplement: Supplementary file 1 [file emmm0007-1366-sd1.pdf]

APPENDIX

Table of Contents

Appendix Figure S1 page 2

Appendix Figure S2 page 3

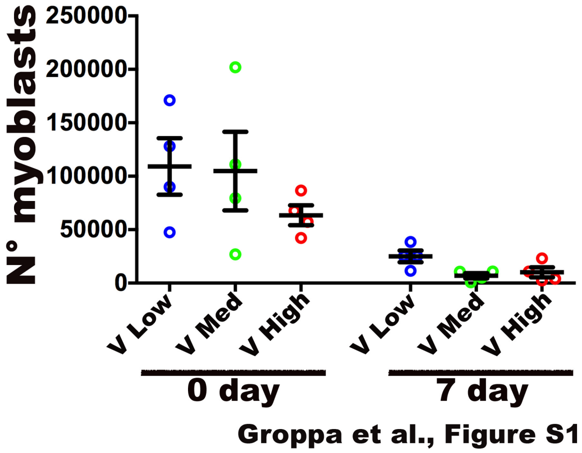

Appendix Figure S1. Similar engraftment of VEGF-expressing myoblast clones. VEGF myoblasts clones were injected in the leg skeletal muscles of SCID mice, which were sacrificed immediately (0 day) or 7 days later. Muscles were collected and the number of myoblasts present in the tissues was measured by qRT-PCR quantification of the retroviral β-gal sequence integrated in genomic DNA. All myoblast populations displayed similar engraftment after the expected significant cell loss over the first 7 days. Data represent individual values, with mean±SEM (n=4). Data were subjected to 1-way ANOVA with Bonferroni multiple comparisons test, after data normalization by logarithmic transformation, and no significant differences were detected.

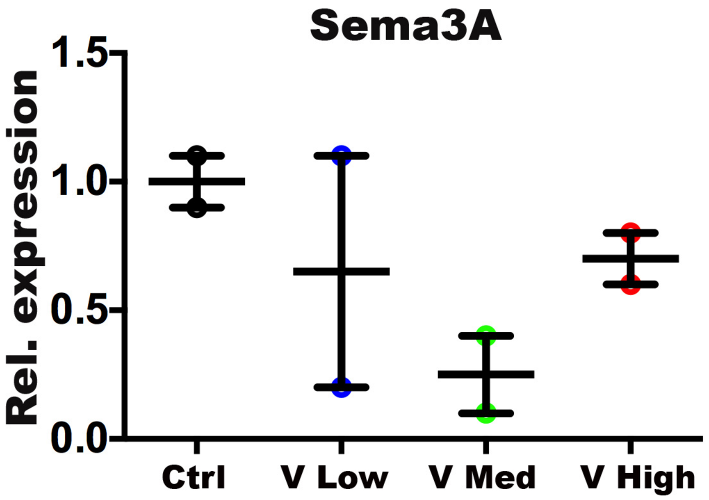

Groppa et al., Figure S2

Appendix Figure S2. Sema3A expression by myoblasts does not correlate with VEGF expression levels. Sema3A relative expression was quantified on total RNA from V Low, V Med, V High and control (Ctrl) myoblasts cultured *in vitro* and expressed as fold-change vs that of control cells. Data represent individual values, with mean±SEM (n=2). Data were subjected to 1-way ANOVA with Bonferroni multiple comparisons test, after data normalization by logarithmic transformation, and no significant differences were detected.
