# Supplementary material for: VEGF dose regulates vascular stabilization through Semaphorin3A and the Neuropilin-1+ monocyte/TGF-β1 paracrine axis
Source: EMBO Mol Med. 2015 Sep 7;7(10):1366–84. doi: 10.15252/emmm.201405003 (PMC4604689; doi:10.15252/emmm.201405003)
Supplement: Supplementary file 2 [file emmm0007-1366-sd2.pdf]

Expanded View Figures

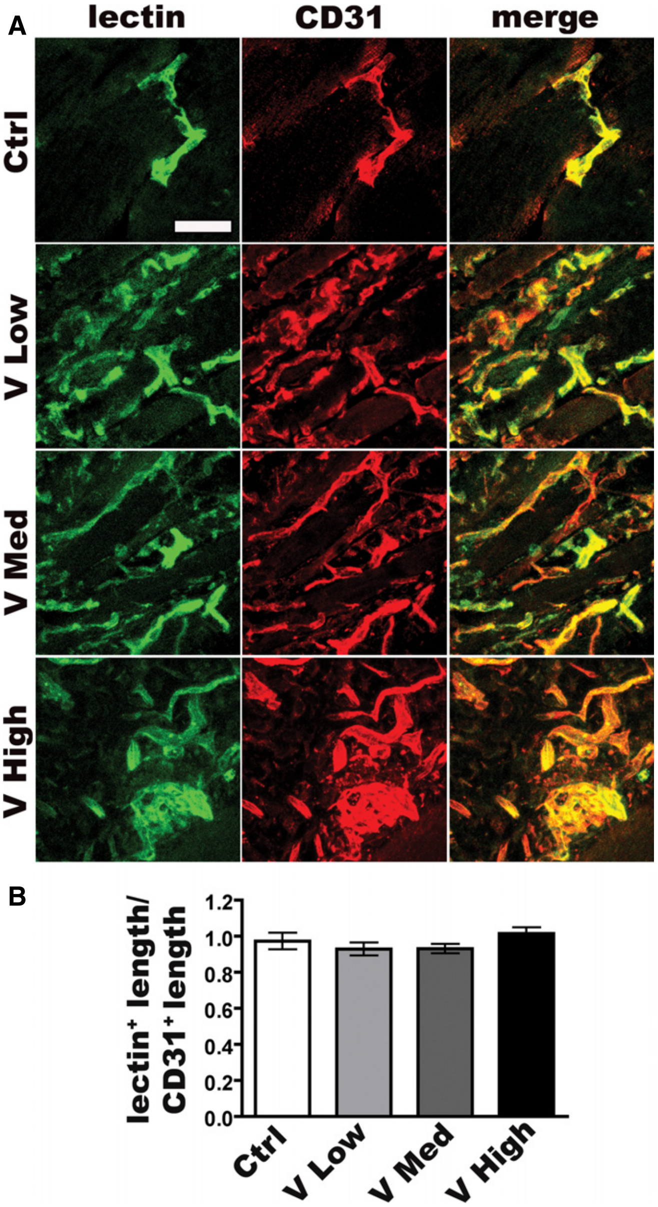

Figure EV1. Vessels induced by different VEGF doses are similarly perfused.

Mice received intravenous injections of FITC-lectin 2 weeks after implantation of myoblast clones expressing different VEGF levels.

A Frozen sections of limb muscles were immunostained for CD31 (endothelium, in red) and perfused structures were visualized by FITC-lectin co-localization (in green). Scale bar = 50 μm.

B Quantification of the perfusion index (lectin-positive vessel length/CD31-positive vessel length) showed that both normal capillaries and aberrant structures induced by the different VEGF doses were similarly perfused. Data represent the mean ± SEM of individual images (*n*) acquired from three muscles/group: Ctrl, $n = 3$; V Low, $n = 8$; V Med, $n = 8$; V High, $n = 7$. Data were subjected to Kruskal–Wallis analysis with Dunn's multiple comparisons test and no significant differences were detected.

Source data are available online for this figure.

 © 2015 The Authors

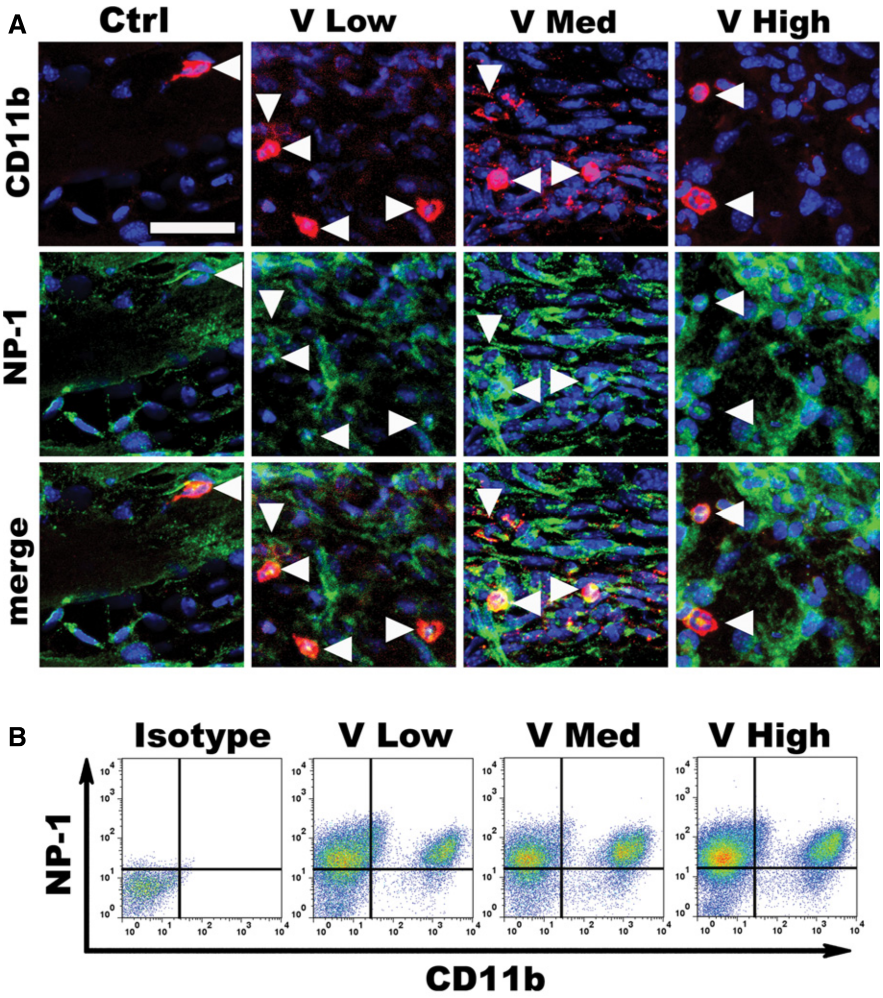

Figure EV2. Mononuclear cells recruited to the sites of VEGF-induced neovascularization express both CD11b and NRP1.

A Immunofluorescence staining for CD11b (in red) and NRP1 (in green) on frozen sections from muscles 1 week after implantation of V Low-, V Med-, and V High-expressing myoblast clones or control cells (Ctrl). Essentially all CD11b-positive cells also expressed NRP1. Nuclei positive for NRP1, but not for CD11b, belong to endothelial cells. Scale bar = 25 μm.

B Flow cytometry analysis confirmed that CD11b-positive monocytes isolated from the muscles implanted with VEGF-expressing cells were also NRP1-positive. The panels show representative data from two independent experiments.

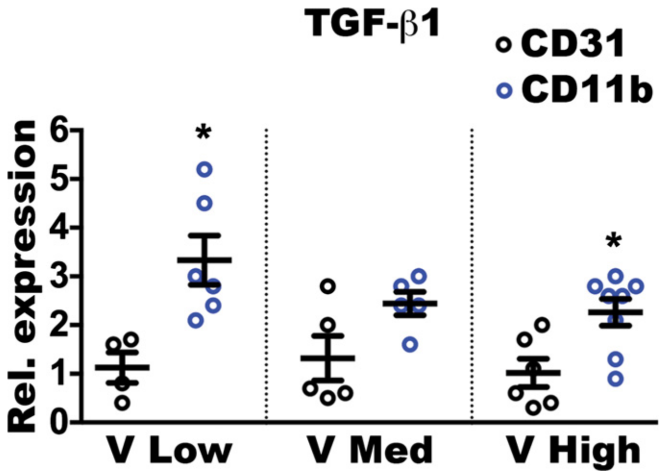

Figure EV3. *Ex vivo* purified NEM express higher levels of TGF-β1 than endothelial cells.

TGF-β1 gene expression was quantified on NEM (CD11b) and endothelial cells (CD31) FACS-purified from muscles 1 week after implantation with V Low-, V Med-, and V High-expressing myoblast clones. The expression in CD11b-positive cells is shown as fold-change versus that in endothelial cells. Data represent individual values, with mean ± SEM (n = 4–7); $^*P < 0.05$ by t-test with Welch's correction: V Low— CD11b versus CD31 P = 0.0315; V High—CD11b versus CD31 P = 0.0281.

Source data are available online for this figure.

© 2015 The Authors

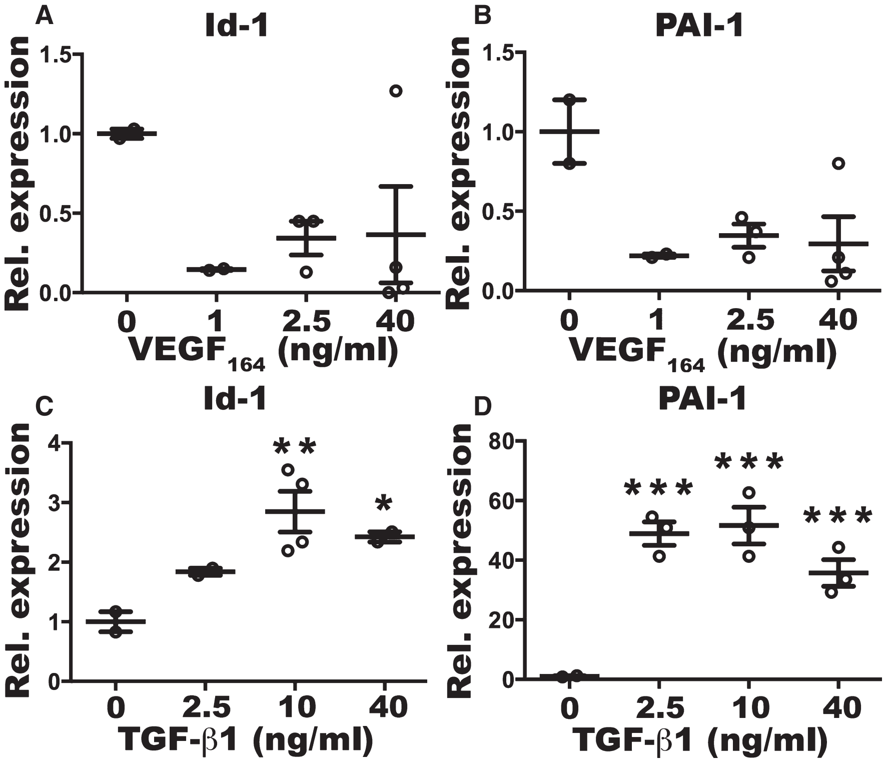

Figure EV4. Expression of the TGF-β1 downstream genes Id-1 and PAI-1 is not stimulated by VEGF.

A–D Mouse aortic endothelial cells were stimulated *in vitro* with increasing doses of recombinant $VEGF_{164}$ or TGF-β1 for 24 h. The expression of Id-1 and PAI-1 was quantified by qRT–PCR and expressed as fold-change versus that of non-stimulated cells. Increasing VEGF doses did not significantly change Id-1 (A) and PAI-1 expression (B), whereas TGF-β1 upregulated both (C, D). Data represent individual values, with mean ± SEM (n = 2–4); *$P < 0.05$, **$P < 0.01$, and ***$P < 0.001$ by one-way ANOVA with Bonferroni multiple comparisons test, after data normalization by logarithmic transformation; Id-1 and PAI-1 expression upon VEGF stimulation: no significant differences were detected; Id-1 expression upon TGF-β1 stimulation: 10 versus 0 $P = 0.0058$; 0 versus 40 $P = 0.0247$; PAI-1 expression upon TGF-β1 stimulation: all comparisons versus 0 $P < 0.0001$.

Source data are available online for this figure.

 © 2015 The Authors
