# Supplementary material for: VEGF dose regulates vascular stabilization through Semaphorin3A and the Neuropilin-1+ monocyte/TGF-β1 paracrine axis
Source: EMBO Mol Med. 2015 Sep 7;7(10):1366–84. doi: 10.15252/emmm.201405003 (PMC4604689; doi:10.15252/emmm.201405003)
Supplement: Supplementary file 3 [file emmm0007-1366-sd3.pdf]

EMBO Molecular Medicine

VEGF dose regulates vascular stabilization through Semaphorin3A and the Neuropilin-1+ monocyte/TGF-β1 paracrine axis

Elena Groppa, Sime Brkic, Emmanuela Bovo, Silvia Reginato, Veronica Sacchi, Nunzia Di Maggio, Manuele G. Muraro, Diego Calabrese, Michael Heberer, Roberto Gianni-Barrera and Andrea Banfi

Corresponding author: Andrea Banfi, Basel University Hospital

Review timeline:

Submission date:	28 December 2014
Editorial Decision:	17 February 2015
Revision received:	19 June 2015
Editorial Decision:	06 July 2015
Accepted:	17 July 2015

Transaction Report:

(Note: With the exception of the correction of typographical or spelling errors that could be a source of ambiguity, letters and reports are not edited. The original formatting of letters and referee reports may not be reflected in this compilation.)

Editor: Roberto Buccione

1st Editorial Decision 17 February 2015

Thank you for the submission of your manuscript to EMBO Molecular Medicine. We are sorry that it has taken longer than usual to get back to you on your manuscript. In this case we experienced unusual difficulties in securing three appropriate reviewers and then obtaining their evaluations in a timely manner. Further to this, I wished to discuss further with my colleagues.

As you will see, the Reviewers find many merits in your manuscript but raise significant issues. I will not dwell into much detail, as their comments are thorough. I would like, however, to highlight a few main points.

All three Reviewers clearly agree that your manuscript suffers from a lack of sufficient mechanistic insight in general and specifically concerning which cells secrete Sema3A under VEGF stimulation, how the monocytes are recruited, the specific roles of TGFbeta1/angiopoietin 2 and what causes the high vs. low VEGF switch. The second major point is that Reviewers 2 and 3 clearly agree that the translational value of the manuscript is currently hampered by the lack of a convincing, more pre-clinical grade model.

In conclusion, while publication of the paper cannot be considered at this stage, given the potential interest of your findings and the fact that the Reviewers, although highly critical, did find your work interesting, we have decided to give you the opportunity to address the above concerns.

We are thus prepared to consider a substantially revised submission, with the understanding that the Reviewers' concerns must be addressed with additional experimental data where appropriate and that acceptance of the manuscript will entail a second round of review. Although we would not ask you to address the entirety of the mechanistic concerns, progress should be made in those respects

and we would need you to show convincing translational relevance by using appropriate models as suggested.

I understand that if you do not have the required data available at least in part, to address the above might entail a significant amount of time, additional work and experimentation and might be technically challenging, I would therefore understand if you chose to rather seek publication elsewhere at this stage. Should you do so, we would welcome a message to this effect.

Please note that it is EMBO Molecular Medicine policy to allow a single round of revision only and that, therefore, acceptance or rejection of the manuscript will depend on the completeness of your responses included in the next, final version of the manuscript.

As you might know, EMBO Molecular Medicine has a "scooping protection" policy, whereby similar findings that are published by others during review or revision are not a criterion for rejection. However, I do ask you to get in touch with us after three months if you have not completed your revision, to update us on the status. Please also contact us as soon as possible if similar work is published elsewhere.

Finally, please note that EMBO Molecular Medicine now requires a complete author checklist (http://embomolmed.embopress.org/authorguide#editorial3) to be submitted with all revised manuscripts. Provision of the author checklist is mandatory at revision stage; The checklist is designed to enhance and standardize reporting of key information in research papers and to support reanalysis and repetition of experiments by the community. The list covers key information for figure panels and captions and focuses on statistics, the reporting of reagents, animal models and human subject-derived data, as well as guidance to optimise data accessibility.

***** Reviewer's comments *****

Referee #1 (Comments on Novelty/Model System):

This is an interesting manuscript describing a novel mechanisms leading to vessel stabilisation/destabilization by different VEGF doses.

Referee #1 (Remarks):

The manuscript by E. Groppa and collaborators reports that the differential induction of Semaphorin 3A (Sema3A) by VEGF, under certain conditions, explains why low doses of VEGF induce mature blood vessel formation while high doses of the same factor instead result in vessel deregulation. This characteristics has been reported by many investigator over the last 15 years, including the authors themselves, but has never found a convincing molecular explanation. Here the authors take advantage of monoclonal cell populations expressing defined levels of VEGF and correlate vessel stabilization with the capacity of Sema3A to attract a recently described population of monocytes (NEMs) displaying the Neuropilin 1 Sema3A receptor and secreting factors causally determining vessel stabilization.

The message of this manuscript is novel, the experiments are overall well conducted and the results are sound. A few issues would however deserve additional attention before publication.

Based on the correlative observations reported by the manuscript, a major advancement would be the understanding of which are the cells that, under the VEGF stimulus, secrete Sema3A and which is the pathway leading to this process. This reviewer appreciates that this would require considerable experimental work, in addition to that already shown in the currently displayed 8 figures. However, the authors should at least amend their discussion to take this issue into account.

Along the same line, by which mechanism does systemic Sema3A administration cause local recruitment of NEMs? Are NEMs cells also found in muscles not expressing VEGF and in the circulation?

Finally, some of the Figures can be improved. For example, Figure 2 could be moved to the Supplementary material. Figure 3A could include a higher magnification image, showing whether

the intense staining observed in the interstitium corresponds to secreted Sema3A or NEMs recruited by this factor.

Referee #2 (Comments on Novelty/Model System):

I assume that the authors aim to systematically study possibilities to improve angiogenic gene therapy in e.g. patients with leg ischemia. With this in mind, myoblast transplantation does not appear to be the first choice. I am not familiar enough with the model to know how much of the transplant is incorporated and what one can achieve functionally in the best scenario by using this method. A truly translational model would perhaps not employ cultured myoblasts and tedious selection of cells with very defined levels of growth factor secretion, but instead viral gene delivery, ultimately with promoters that can be switched on and off. Another useful model would perhaps be the tet-regulated VEGF model used in other tissues by Eli Keshet and collaborators (a recent example: http://www.ncbi.nlm.nih.gov/pubmed/24166715). Furthermore, one should perhaps avoid using immunodeficient mice as the patients in need of such vessels most commonly are not immunosuppressed. Furthermore, even considering basic mechanisms of vessel stabilization in vivo, factors such as Sema 3A and TGFß are likely to transduce signals via additional cells of the adaptive immune response, thus an immunodeficient mouse background should be avoided.

Referee #2 (Remarks):

Clinical attempts to use angiogenic therapies, especially those based on use of VEGF have not been fully successful in part due to angioma formation and instability of the new formed blood vessels. The manuscript by Groppa et al. analyzes how VEGF dosage affects on the stability of the vessels generated in a cell and gene therapy model where the authors use retrovirally transduced mouse myoblasts that stably express specific low, medium and high VEGF doses. These cells were implanted into leg skeletal muscles of adult SCID mice. High-VEGF clones induced mainly aberrant structures, as reported previously by the same group, whereas low-VEGF clones induced normal vessels, which became VEGF-independent in about 10 days. The authors show that the expression levels of Pdgfb, Ang1 and Ang2 were similar in all three groups, whereas Sema3a and Tgfß1 were significantly more upregulated in the low-VEGF group where vessels were best stabilized in comparison to the other two groups. The authors explain the stabilization effect observed in the low-VEGF group by Sema3a-dependent recruitment of NEM (NP-1 expressing monocytes), which then produce an excess of TGFß1 for vessel stabilization.
The model as used in immunodeficient mice is not the best one available for studies of proangiogenic therapy and the advance presented with the model is incremental. Furthermore, while TGFß upregulation may be necessary for vessel stabilization, this conclusion is based on detection of some typical TGFß induced responses and on extrapolated deductions from other contexts, which have not been rigorously tested by deleting TGFß in the system used.
These cricisms need to be addressed before the manuscript can be reconsidered for EMM.
Below are my detailed
1. I am not familiar enough with the myoblast transplantation model to know how much of the transplant is incorporated and what one can achieve functionally in the best scenario by using this method. A truly translational model would perhaps not employ cultured myoblasts and tedious selection of cells with very defined levels of growth factor secretion, but instead viral gene delivery, ultimately with promoters that can be switched on and off. Another useful model would perhaps be the tet-regulated VEGF model used in other tissues by Eli Keshet and collaborators (a recent example: http://www.ncbi.nlm.nih.gov/pubmed/24166715). Furthermore, one should perhaps avoid using immunodeficient mice as the patients in need of such vessels most commonly are not immunosuppressed. Furthermore, even considering basic mechanisms of vessel stabilization in vivo, factors such as Sema 3A and TGFß are likely to transduce signals via additional cells of the adaptive immune response, thus an immunodeficient mouse background should be avoided. In several therapeutic conditions, arteriogenesis would be more important than angiogenesis, thus vessel arterialization (smooth muscle coated vessels) and tissue perfusion (e.g. ultrasound-microbubble based analysis) could be quantified to reinforce the translational value of this work. It is important to remember that shear stress is a major factor for eventual vessel stabilization. Maybe the authors could comment on these aspects in their discussion.

2. Previous studies have shown that Sema3a recruits NEMs in the tumor setting. In vitro, NEMs were also chemoattractive for smooth muscle cells, but the factor(s) have not been identified (study of A. Carrer et al. (2012)). Here Figure 1 presents essentially the same result as Figure 7 of the 2004 JCI publication by Ozama, Banfi et al. (http://www.ncbi.nlm.nih.gov/pubmed/14966561), but now with additional NG2 and SMA staining, and the authors mention that high VEGF doses were associated with recruitment of SMA expressing perivascular cells instead of the NG2 expressing cells (p. 7). Perhaps the authors could comment on smooth muscle cell chemoattractants that could be involved in their model.
3. The manuscript presents evidence indicating that TGFß and SMAD2/3 target PAI-1 levels correlate with vessel stabilization, but definitive evidence that TGFß is involved is not included. Even the reference provided for the vascular stabilization effect of TGFß (Goumans et al., 2002) mentions that "..induction of plasminogen activator inhibitor-1 by activated ALK5 may contribute to the TGF-beta-induced maturation of blood vessels." While inhibition of Sema3a signaling by the NRP1 blocking antibody is a nice experiment (assuming that other semaphorins are not involved), but none of the experiments directly addresses the role of TGFß1 or angiopoietin2 by blocking antibodies.
4. One gets the impression from Figure 3 that even in the SCID mice, there is a massive recruitment of (inflammatory?) cells by the high dose of VGEF. If this is the case, there should be a plethora of factors that are made by the inflammatory cells and differ between the low and high VEGF doses. These would be best analyzed by global transcriptomic profiling instead of the candidate gene approach. Furthermore, when the relative levels of gene expression are presented, these should preferably be from multiple time points, considering the complex dynamics of the model. Some supplementary low-power images of HE sections that give a general view of the transplant with surrounding normal muscle would certainly help the reviewer the evaluation of the translational potential of this model.
5. The mechanistic part requires some clarification. For instance, the authors show that Sema3a was upregulated 5-fold in skeletal muscle of low-VEGF group (qPCR analysis of RNA) - Figure 3A. At the same time, isolated ECs did not show any Sema3a upregulation at any VEGF dose, but instead very moderate downregulation (2-fold at highest VEGF dose) - Figure 7A. The authors notice this discrepancy in the Discussion and offer a hypothetic explanation, in which they suggest a feedback loop involving EC-produced Ang2 and the ability of TGFβ1 to induce Sema3a expression in isolated ECs. This explanation, however, is questionable considering that (A) Ang2 expression was found at about the same level (in treated skeletal muscle) in all groups; and (B) The highest VEGF dose (Figure 7A) caused twofold downregulation, whereas TGFβ1 induced 7-12-fold upregulation of Sema3a. This means that inhibition of Sema3a caused by high VEGF dose was not enough to prevent upregulation of Sema3a caused by TGFβ1, at least in vitro. The source of Sema3a is not obvious, but it doesn't seem to be endothelial cells, or at least the evidence is not sufficient for such conclusion. Finally, the last chapter indicating that Sema3A promotes vascular stabilization is somewhat confirmatory of earlier results in the tumor setting.

Referee #3 (Remarks):

Groppa et al investigated the role of Semaphorin-3A and neuropilin-1 in VEGF mediated vessel stabilization. They found that low VEGF induced vessel stabilization, whereas high VEGF was incapable of producing stable vessels. Mechanistically they found that low VEGF induced recruitment of circulating cells involving endothelial Sema3A, acting on neuropilin-1 receptors on monocytes. They furthermore showed that the recruited cells produced TGFbeta which subsequently activated Smad signalling in endothelial cells. Moreover, TGFbeta stimulated Sema3A production by endothelial cells thus providing a positive feedback loop. Administering Sema3A to the high VEGF treatment group restored vessel stabilization. This is an interesting paper with potential impact for treatment strategies in ischemic cardiovascular diseases or normalization of tumor vasculatures. However, there are also some issues that need to be addressed and they are listed below:

It is unclear how vessel stabilization is defined. The authors state: stabilization is a functional property of persistence. In the experimental setting the authors determine length density after system treatment with VEGF-trap. What is the rationale for selecting this approach? Several reports suggest

that VEGF-trap affects local hemodynamics, by scavenging VEGF that acts as a local vasodilator involving nitric oxide. Local hemodynamic factors like blood flow affect vessel regression or maintenance. Is this considered in the vessel stabilization concept? VEGF itself is furthermore considered to be important for "lumen diameter growth" of capillaries and small arterioles - is this inhibited by VEGF-trap? Did the authors check VEGF receptor signalling upon VEGF-trap?

For the injected myoblasts: did the authors observe differences between the Tibialis and Gastrocnemius muscle? From the images provided in the figures it is not clear which muscle was used.

Figure 1: this figure needs to be clarified. Please provide a macroscopic image or illustration showing where the myoblasts were injected, and which muscle was analyzed (Tibialis / Gastro). It is difficult to see the skeletal muscle organization on the images provided; how are the sections projected (transverse/longitudinal)? In a normal histological section, skeletal muscle fibres are surrounded by several capillaries and only a few small arterioles and venules. Can the authors indicate these vascular compartments in their sections. Please provide an image of a normal, uninjected control, and images of the "site remote of myoblast implementation, the area with VLD 15 mm/mm2). Can the authors provide evidence showing that VEGF-trap actually reached the implanted myoblasts, and reduced VEGF availability and/or signalling in endothelial cells? Are the effects comparable between the arteriolar and venular part of the vascular bed investigated? VEGF has been linked to specifying arterial identity in growing capillaries - is this inhibited upon VEGF-trap in this setting and would it influence the "stabilization" measurements?

Why do low and medium VEGF levels induce pericyte recruitment and high VEGF levels VSMC? It is not clear why recruitment of VSMC, as seen in high VEGF, didn't result in a stabilized vessel. In the context of vessel stabilization, what is the functional difference between pericytes and VSMC recruitment? Skeletal muscle arterioles and venules are surrounded by VSMC and they are usually considered "stable". Can the authors clarify this issue?

In some settings like the nervous system and sprouting blood vessels, Semaphorin-3A is considered to act as a repulsive guidance cue involving Neuropilin-1 and Plexin receptors. What turns Sema3A into an attractive agent with respect to recruitment of neuropilin-1 positive bone marrow derived cells? What percentage of the total monocytes/macrophage population expresses neuropilin-1? Neuropilin-1 is also expressed by growing sprouts and arterioles (it is considered an arterial marker). Is there any evidence for an endothelial cell-autonomous, Sema3A-neuropilin-1 autocrine loop contributing to the monocyte, or pericyte recruitment upon grafting of the VEGF producing myoblasts? In the collateral arteriogenesis setting, VEGF has been shown to promote influx of macrophages/monocytes into growing and expanding collateral arteries. In the myoblast setting, high VEGF levels associate with reduced recruitment of monocytes; can the authors explain this difference?

Figure 3: can you indicate the site where the myoblasts were implanted?
Figure E2: the variance in the Sema3a measurements is too large to make any statement. This experiment needs to be repeated (compare the error bar in VEGF low with VEGF high). To provide direct evidence showing that the Sema3A - monocyte axis is essential, the authors should consider depleting the mouse macrophage / monocyte population pharmacologically or genetically and analyse vessel stabilization upon low VEGF treatment.

With respect to stabilization: what is causing the switch between low and high dose VEGF. Do these results imply that the amount of VEGF-R2 phosphorylation is the key regulator? Can the authors provide data indicating how this switch is mechanistically regulated?

For the rescue experiments: the authors should show that the injected Sema3A protein activated Neuropilin-1 signalling in the monocyte population. In the same line: where are the neuropilin-1 receptors expressed in this setting (endothelial cells, monocytes,VSMC/pericytes/muscle tissue) - is it possible that Sema3A directly acts on pericytes or smooth muscle cells? Surprisingly, Sema3A treatment didn't affect the amount of vascular growth (Figure 8). However, monocytes are considered to stimulate angiogenesis, promote fusion between angiogenic sprouts and arteriogenesis. How do the authors explain that on the one hand Sema3A promotes influx of monocytes, but on the other hand doesn't affect angiogenesis?

The authors may want to test their findings in an ischemia model - for example the femoral artery ligation assay. If a cocktail of low VEGF and Sema3A indeed makes functional perfused vessels that can carry blood flow to the compromised regions, then the findings may have a broad impact.

1st Revision - authors' response 19 June 2015

***** Reviewer's comments *****

Referee #1 (Comments on Novelty/Model System):

This is an interesting manuscript describing a novel mechanisms leading to vessel stabilisation/destabilization by different VEGF doses.

We would like to thank the Reviewer for his appreciation.

Referee #1 (Remarks):

The manuscript by E. Groppa and collaborators reports that the differential induction of Semaphorin 3A (Sema3A) by VEGF, under certain conditions, explains why low doses of VEGF induce mature blood vessel formation while high doses of the same factor instead result in vessel deregulation. This characteristic has been reported by many investigators over the last 15 years, including the authors themselves, but has never found a convincing molecular explanation. Here the authors take advantage of monoclonal cell populations expressing defined levels of VEGF and correlate vessel stabilization with the capacity of Sema3A to attract a recently described population of monocytes (NEMs) displaying the Neuropilin 1 Sema3A receptor and secreting factors causally determining vessel stabilization.

The message of this manuscript is novel, the experiments are overall well conducted and the results are sound. A few issues would however deserve additional attention before publication.

1. Based on the correlative observations reported by the manuscript, a major advancement would be the understanding of which are the cells that, under the VEGF stimulus, secrete Sema3A and which is the pathway leading to this process. This reviewer appreciates that this would require considerable experimental work, in addition to that already shown in the currently displayed 8 figures. However, the authors should at least amend their discussion to take this issue into account.

We would like to thank the Reviewer for raising these important points, which we have addressed with new experiments, reported in new Figures 2C-D and 6D-E: 1) the identity of Sema3A-expressing cells under VEGF stimulation *in vivo* was investigated by fluorescence *in situ* hybridization (Figure 2C-D) and the results show that greater than 90% of the Sema3A-expressing cells are endothelial, while conversely 86% of the endothelial cells in areas of VEGF expression produce Sema3A; 2) a blocking experiment by systemic treatment of animals with an anti-TGF-β1 antibody (Figure 6D-E) showed that Sema3A production *in vivo* is not directly stimulated by VEGF, but requires TGF-β1 signaling, as both Sema3A upregulation and NEM recruitment were abolished by TGF blockade despite VEGF expression.

2. Along the same line, by which mechanism does systemic Sema3A administration cause local recruitment of NEMs? Are NEMs cells also found in muscles not expressing VEGF and in the circulation?

We realized that the provided description of the Sema3A delivery was a source of misunderstanding and we have now clarified the protocol for treatment with recombinant Sema3A in the methods (page 24, last paragraph) and Results (page 13, second paragraph). Indeed treatment was not systemic, but localized by intramuscular injection: the 2 investigated doses were expressed as mg/kg of average tissue weight. Therefore, each muscle of each animal received a fixed dose calculated on the average weight of the *Tibialis anterior* or *Gastrocnemius* muscles that were treated, based on measurements of the muscles harvested for several other experiments (60 mg for

Tibialis and 120 mg for *Gastrocnemius*, corresponding to 6 or 60 µg and 12 or 120 µg of recombinant Sema3A-Fc, respectively for the 2 doses tested).

3. Finally, some of the Figures can be improved. For example, Figure 2 could be moved to the Supplementary material.

We agree with the Reviewer and the Figure has now been moved to the Expanded View section (new Figure EV1).

4. Figure 3A could include a higher magnification image, showing whether the intense staining observed in the interstitium corresponds to secreted Sema3A or NEMs recruited by this factor.

We find it difficult to associate the antibody stain to a specific cell type in immunohistochemistry, as the secreted Sema3A protein diffuses somewhat in the matrix. However, in order to more rigorously address the source of Sema3A production in the areas of VEGF-induced angiogenesis, we have performed *in situ* hybridization experiments, finding that greater than 90% of the Sema3A-producing cells are endothelial (Figure 2C-D).

Referee #2 (Comments on Novelty/Model System):
I assume that the authors aim to systematically study possibilities to improve angiogenic gene therapy in e.g. patients with leg ischemia. With this in mind, myoblast transplantation does not appear to be the first choice. I am not familiar enough with the model to know how much of the transplant is incorporated and what one can achieve functionally in the best scenario by using this method. A truly translational model would perhaps not employ cultured myoblasts and tedious selection of cells with very defined levels of growth factor secretion, but instead viral gene delivery, ultimately with promoters that can be switched on and off. Another useful model would perhaps be the tet-regulated VEGF model used in other tissues by Eli Keshet and collaborators (a recent example: http://www.ncbi.nlm.nih.gov/pubmed/24166715).

Please see response to point 1a below.

Furthermore, one should perhaps avoid using immunodeficient mice as the patients in need of such vessels most commonly are not immunosuppressed. Furthermore, even considering basic mechanisms of vessel stabilization in vivo, factors such as Sema 3A and TGFb; are likely to transduce signals via additional cells of the adaptive immune response, thus an immunodeficient mouse background should be avoided.

Please see response to point 1b below.

Referee #2 (Remarks):
Clinical attempts to use angiogenic therapies, especially those based on use of VEGF have not been fully successful in part due to angioma formation and instability of the new formed blood vessels. The manuscript by Groppa et al. analyzes how VEGF dosage affects on the stability of the vessels generated in a cell and gene therapy model where the authors use retrovirally transduced mouse myoblasts that stably express specific low, medium and high VEGF doses. These cells were implanted into leg skeletal muscles of adult SCID mice. High-VEGF clones induced mainly aberrant structures, as reported previously by the same group, whereas low-VEGF clones induced normal vessels, which became VEGF-independent in about 10 days. The authors show that the expression levels of Pdgfb, Ang1 and Ang2 were similar in all three groups, whereas Sema3a and TGFb1 were significantly more upregulated in the low-VEGF group where vessels were best stabilized in comparison to the other two groups. The authors explain the stabilization effect observed in the low-VEGF group by Sema3a-dependent recruitment of NEM (NP-1 expressing monocytes), which then produce an excess of TGFb1 for vessel stabilization.
The model as used in immunodeficient mice is not the best one available for studies of proangiogenic therapy and the advance presented with the model is incremental. Furthermore,

while TGFb upregulation may be necessary for vessel stabilization, this conclusion is based on detection of some typical TGFb induced responses and on extrapolated deductions from other contexts, which have not been rigorously tested by deleting TGFb in the system used.
These cricisms need to be addressed before the manuscript can be reconsidered for EMM.
Below are my detailed
1a. I am not familiar enough with the myoblast transplantation model to know how much of the transplant is incorporated and what one can achieve functionally in the best scenario by using this method. A truly translational model would perhaps not employ cultured myoblasts and tedious selection of cells with very defined levels of growth factor secretion, but instead viral gene delivery, ultimately with promoters that can be switched on and off. Another useful model would perhaps be the tet-regulated VEGF model used in other tissues by Eli Keshet and collaborators (a recent example: http://www.ncbi.nlm.nih.gov/pubmed/24166715).
1b. Furthermore, one should perhaps avoid using immunodeficient mice as the patients in need of such vessels most commonly are not immunosuppressed. Furthermore, even considering basic mechanisms of vessel stabilization in vivo, factors such as Sema 3A and TGFb are likely to transduce signals via additional cells of the adaptive immune response, thus an immunodeficient mouse background should be avoided.

We agree with the Reviewer that a translational model would require a clinically relevant method of VEGF delivery and an immunocompetent background, while monoclonal populations of transduced myoblasts were employed as a unique model to precisely control the microenvironmental dose of VEGF expression and perform dose-dependent studies. We have therefore performed experiments with adenoviral (AV) delivery, as suggested, in immunocompetent C57/Bl6 mice and results are shown in new Figure 7D-F. Under these conditions, more reflective of a clinical setting, the immune response to the AV vector leads to robust, but transient VEGF expression and efficient angiogenesis, which is initially induced by 1 week, completely regresses after the AV vector is eliminated during the first 10 days. However, we found that local Sema3A treatment with only 2 intramuscular doses, injected during the first week, is sufficient to increase NEM recruitment also in immunocompetent animals and accelerate the stabilization of the induced vasculature, preventing its regression by 3 weeks. These data in immunocompetent animals confirm the results obtained in the immunodeficient SCID model on one hand, and support the translational potential of our findings on the other.

1c. In several therapeutic conditions, arteriogenesis would be more important than angiogenesis, thus vessel arterialization (smooth muscle coated vessels) and tissue perfusion (e.g. ultrasound-microbubble based analysis) could be quantified to reinforce the translational value of this work. It is important to remember that shear stress is a major factor for eventual vessel stabilization. Maybe the authors could comment on these aspects in their discussion.

The Reviewer raises an interesting point, which we have now addressed in the Discussion (page 17, third paragraph). Indeed, we have previously investigated the effects of VEGF dose on blood flow with the same monoclonal myoblast populations used here and we found that the V-med conditions led to the highest increase in tissue perfusion (measured by the gold-standard method of microsphere entrapment; von Degenfeld et al. FASEB J. 2006), although stabilization is actually fastest in the V-low conditions. Further, it should be underlined that the vessels induced by both low and medium VEGF levels displayed the morphology of normal capillaries and were similarly associated with SMA-negative pericytes (Figure 1 and page 7, first paragraph), while aberrant structures induced by high VEGF, which were covered by smooth-muscle cells, actually did not stabilize and remained exquisitely sensitive to VEGF blockade (page 6, first paragraph).

2a. Previous studies have shown that Sema3a recruits NEMs in the tumor setting. In vitro, NEMs were also chemoattractive for smooth muscle cells, but the factor(s) have not been identified (study of A. Carrer et al. (2012)). Here Figure 1 presents essentially the same result as Figure 7 of the 2004 JCI publication by Ozawa, Banfi et al. (http://www.ncbi.nlm.nih.gov/pubmed/14966561), but now with additional NG2 and SMA staining,…

We have now clarified in the Introduction (page 3, first paragraph) the relation between previous results by us and others (as shown, for example in Fig. 7 of our previous paper by Ozawa et al, JCI 2004) and those reported here. In fact, we previously reported only that normal new vessels induced by low VEGF were stable by 4 weeks, while the aberrant structures induced by high levels never stabilized. Here, instead, we carefully investigated the VEGF dose-dependent kinetics of stabilization with 2 early time points at 10 and 17 days after induction and with 3 different VEGF doses (2 of which induce equally normal angiogenesis, but distinct stabilization kinetics), in order to define the molecular mechanisms involved.

2b. …and the authors mention that high VEGF doses were associated with recruitment of SMA expressing perivascular cells instead of the NG2 expressing cells (p. 7). Perhaps the authors could comment on smooth muscle cell chemoattractants that could be involved in their model.

We have now discussed (page 17, second paragraph) the possible link, suggested by the Reviewer, between the ability of NEM-produced paracrine factors to attract smooth muscle cells in vitro, described in the referenced work by the group of Mauro Giacca, with the switch in mural cell coverage (pericytes vs smooth muscle) observed between normal capillaries induced by low VEGF and aberrant structures induced by high VEGF. However, our data suggest that it is unlikely that NEM-derived chemoattractants may play a role in the observed transition in mural cell coverage between VEGF doses, as the condition where smooth muscle cells are present (high VEGF) corresponds to the minimum amount of NEM recruitment (Figure 3). On the other hand, here we investigated the stabilization kinetics of capillary networks, which are associated exclusively with SMA-negative pericytes in skeletal muscle and not with smooth-muscle cells. In this regard, we found that, while the pericyte-recruiting factor PDGF-BB was upregulated after VEGF delivery, its tissue levels did not change with VEGF dose (Figure 2A): consistently, vessels induced by both low and medium VEGF doses were similarly mature (associated with SMA-negative pericytes), yet stabilized with different kinetics, suggesting that vessel stabilization requires further steps that are independent of pericytes.

3. The manuscript presents evidence indicating that TGFb and SMAD2/3 target PAI-1 levels correlate with vessel stabilization, but definitive evidence that TGFb is involved is not included. Even the reference provided for the vascular stabilization effect of TGFb (Goumans et al., 2002) mentions that "…induction of plasminogen activator inhibitor-1 by activated ALK5 may contribute to the TGF-beta-induced maturation of blood vessels." While inhibition of Sema3a signaling by the NRP1 blocking antibody is a nice experiment (assuming that other semaphorins are not involved), but none of the experiments directly addresses the role of TGFb1 or angiopoietin2 by blocking antibodies.

We have now discussed (page 18, first paragraph) the role of PAI in the stabilization of new vessels. Indeed PAI-1 has been shown to mediate TGF-β1-induced vascular stabilization by preventing degradation of the provisional ECM deposited around the new vessel and favoring the establishment of new basal lamina, i.e. PAI-1 is a downstream effector of TGF-β1 signaling and not an independent mechanism. Therefore, the specific upregulation of PAI-1 and phosphorylation of SMAD2/3, which we observed in low VEGF conditions, support the conclusion that the abundant TGF-β1 secreted by recruited NEM preferentially activates the stabilizing signaling pathway in endothelium (Alk5) over the pathway activating proliferation and migration (Alk1-SMAD1/5-Id1). Interestingly, it has been shown that preferential activation of the Alk1 or Alk5 pathway depends on TGF-β1 dose, with signaling switching from Alk1 to Alk5 as TGF-β1 amount increases, consistently with our findings, where the most rapid stabilization is caused by the highest TGF-β1 upregulation. On the other hand, we have now also performed a blocking experiment with an anti-TGF-β1 antibody and found that TGF-β1 inhibition abolishes both Sema3A upregulation and NEM recruitment (Fig. 6D-E). While these results clearly show that TGF-β1 is a key element of a feedback loop between NEM and endothelium sustaining Sema3A expression, they would not allow one to determine that TGF-β1 is specifically responsible for endothelial stabilization, as its blockade disrupts the whole stabilization axis, including NEM recruitment. This would probably require endothelial-specific disruption of Alk5 signaling in an inducible transgenic model (to avoid early

effects during development). However, considering also the already well established role in the literature for TGF-β1 in vascular stabilization, these experiments would probably be too far from the main findings of this work, i.e. that VEGF inhibits vascular stabilization dose-dependently by inhibiting endothelial Sema3A expression and the NEM/TGF-β1 axis, sustained by a novel TGF-β1/Sema3A feedback loop.

4a. One gets the impression from Figure 3 that even in the SCID mice, there is a massive recruitment of (inflammatory?) cells by the high dose of VEGF. If this is the case, there should be a plethora of factors that are made by the inflammatory cells and differ between the low and high VEGF doses. These would be best analyzed by global transcriptomic profiling instead of the candidate gene approach.

We agree that a global transcriptomic profile could provide more wide-ranging information, although with an associated increase in the complexity of result interpretation. Indeed such analyses are being performed for a different ongoing project, but results are not available and go well beyond the scope of the current work. However, we would like to underline that the targeted gene approach we employed has nevertheless yielded a clear hypothesis that we aimed to verify in this manuscript. While a contribution of other pathways and cell types cannot be excluded, our results reported here show that the endothelial Sema3A/NEM/TGF-β1 paracrine loop plays a key role in determining the stabilization rate of VEGF-induced angiogenesis in skeletal muscle, as well as its potential to control this process therapeutically in a clinically-relevant model of adenoviral VEGF delivery in immunocompetent animals (Fig. 7D-F). Additionally, previous analyses we have performed with this model of VEGF-expressing myoblasts over the last decade indicate that the interstitial cells visible in the H&E-counterstained IHC panels shown in Fig. 2B are actually comprised mostly of the injected myoblasts, which have not yet completely fused into myofibers at this stage, and induced vasculature (endothelium and pericytes), with a limited amount of monocytes (Springer et al. Dev. Cell 2000; Springer et al. Mol. Ther. 2003).

4b. Furthermore, when the relative levels of gene expression are presented, these should preferably be from multiple time points, considering the complex dynamics of the model.

We have further clarified the rationale for choosing 7 days as the most relevant time-point for gene expression analysis (page 8, first paragraph): in fact the first differences in stabilization rate were already evident with VEGF-Trap treatment starting at 10 days and we sought to identify the immediately preceding changes. We agree that a kinetic study of gene expression could provide potentially interesting data, but we feel that this kind of information would be branching off in a different direction from the main question addressed here, namely the mechanisms underlying VEGF dose-dependent differences in vascular stabilization, and therefore we focused on the effects of dose at a relevant time-point rather than on time-dependent changes in gene expression.

4c. Some supplementary low-power images of HE sections that give a general view of the transplant with surrounding normal muscle would certainly help the reviewer the evaluation of the translational potential of this model.

We have now clarified in the legend of Figure 2B that the right column of panels represent higher-magnification views of the panels on the left and that these lower-magnification images on the left already capture the majority of the implantation site with some surrounding normal muscle. Naturally, these implantation sites only comprise a limited area of the injected muscle tissue, which limits their validity as a therapeutic approach. However, this approach was chosen to take advantage of the high degree of control over VEGF dose that it affords, which is key to the mechanistic questions addressed here, while the established biological concept was investigated for its potential as a therapeutic strategy in clinically relevant, but uncontrolled, model of adenoviral delivery (Fig. 7D-F) and rigorous preclinical evaluation will require further ad-hoc studies in ischemic models.

5a. The mechanistic part requires some clarification. For instance, the authors show that Sema3a was upregulated 5-fold in skeletal muscle of low-VEGF group (qPCR analysis of RNA) - Figure 3A. At the same time, isolated ECs did not show any Sema3a upregulation at any VEGF dose, but

instead very moderate downregulation (2-fold at highest VEGF dose) - Figure 7A. The authors notice this discrepancy in the Discussion and offer a hypothetic explanation, in which they suggest a feedback loop involving EC-produced Ang2 and the ability of TGFb1 to induce Sema3a expression in isolated ECs. This explanation, however, is questionable considering that (A) Ang2 expression was found at about the same level (in treated skeletal muscle) in all groups; and (B) The highest VEGF dose (Figure 7A) caused twofold downregulation, whereas TGFb1 induced 7-12-fold upregulation of Sema3a. This means that inhibition of Sema3a caused by high VEGF dose was not enough to prevent upregulation of Sema3a caused by TGFb1, at least in vitro.

We agree with the Reviewer that the emerging mechanism suggests several layers of complexity. Therefore, we have now provided further mechanistic insight about the role of TGF-β1 signaling in the regulation of both Sema3A expression and NEM recruitment *in vivo* by performing a pharmacological inhibition of TGF-β1 signaling. Systemic treatment with a specific blocking antibody showed that TGF-β1 signaling is required to sustain both Sema3A expression and NEM recruitment in conditions of low VEGF (Fig. 6D-E), complementing the in vitro results shown in Fig. 6C and proving the *in vivo* function of a novel feedback loop between endothelial Sema3A, NEM recruitment, TGF-β1 production by NEM and further Sema3A expression by endothelium (Fig. 8). The role of initial Ang2 release by activated endothelium remains to be tested in specific genetic models, but we provide a speculation, based on available data, on how similar levels of Ang2 production at all VEGF doses, combined with the direct inhibition of endothelial Sema3A exclusively by high doses of VEGF, may explain the switch between low and high VEGF doses (page 18-19).

Regarding the comparison between the degree of upregulation of Sema3A expression by TGF-β1 and its inhibition by VEGF *in vitro*, these data provide evidence for the positive and negative regulation of endothelial Sema3A by these factors, but we do not feel that too much emphasis should be placed on the quantitative relationships between the *in vitro* experiments, as *in vitro* conditions of isolated proliferating endothelial cells do not accurately reflect the physiological situation of endothelium incorporated in a vascular structure *in vivo*. We have therefore removed the related sentence in the Discussion (page 18, second paragraph).

5b. The source of Sema3a is not obvious, but it doesn't seem to be endothelial cells, or at least the evidence is not sufficient for such conclusion.

We agree that several cell types have the potential to produce Sema3A. Therefore, we have investigated the source of Sema3A production in the areas of VEGF-induced angiogenesis by *in situ* hybridization and we found that greater than 90% of Sema3A-expressing cells are indeed endothelial (Fig. 2C-D), supporting the proposed model of cross-talk between endothelium and NEM through Sema3A and TGF-β1 signaling.

5c. Finally, the last chapter indicating that Sema3A promotes vascular stabilization is somewhat confirmatory of earlier results in the tumor setting.

We have now clarified in the Introduction (page 4, first paragraph) the relationship between previous literature in tumor models and the main scope of our work to determine the role of Sema3A and NEM recruitment to promote new vessel stabilization and VEGF-independence and its therapeutic implications. In fact, in those studies vascular stability was not investigated, as no VEGF-blocking experiment was performed. Rather, in these models Sema3A and NEM recruitment have been shown to inhibit tumor angiogenesis and promote pericyte recruitment to dysfunctional tumor vessels, leading to a reduction in vascular leakage. We have discussed a possible explanation for the consistent lack of anti-angiogenic effect in our experiments at pages 19-20.

Referee #3 (Remarks):
Groppa et al investigated the role of Semaphorin-3A and neuropilin-1 in VEGF mediated vessel stabilization. They found that low VEGF induced vessel stabilization, whereas high VEGF was incapable of producing stable vessels. Mechanistically they found that low VEGF induced

recruitment of circulating cells involving endothelial Sema3A, acting on neuropilin-1 receptors on monocytes. They furthermore showed that the recruited cells produced TGFbeta which subsequently activated Smad signalling in endothelial cells. Moreover, TGFbeta stimulated Sema3A production by endothelial cells thus providing a positive feedback loop. Administering Sema3A to the high VEGF treatment group restored vessel stabilization. This is an interesting paper with potential impact for treatment strategies in ischemic cardiovascular diseases or normalization of tumor vasculatures. However, there are also some issues that need to be addressed and they are listed below:

1. It is unclear how vessel stabilization is defined. The authors state: stabilization is a functional property of persistence. In the experimental setting the authors determine length density after system treatment with VEGF-trap. What is the rationale for selecting this approach? Several reports suggest that VEGF-trap affects local hemodynamics, by scavenging VEGF that acts as a local vasodilator involving nitric oxide. Local hemodynamic factors like blood flow affect vessel regression or maintenance. Is this considered in the vessel stabilization concept? VEGF itself is furthermore considered to be important for "lumen diameter growth" of capillaries and small arterioles - is this inhibited by VEGF-trap? Did the authors check VEGF receptor signalling upon VEGF-trap?

We have clarified the definition of stabilization and the rationale for selecting VEGF abrogation by VEGF-Trap treatment as the experimental approach in the Introduction (page 4, first paragraph) and Results (page 5, first paragraph). The mode of action of VEGF-Trap (Aflibercept) has been extensively studied in a broad body of literature leading up to its characterization and final approval as an anti-angiogenic therapy for colorectal cancer and macular degeneration, showing that VEGF-Trap potently abrogates VEGF signaling through VEGF-Receptors (e.g. Holash et al., PNAS 2002): therefore, we have not repeated measurements of inhibition of VEGF-Receptor phosphorylation after VEGF-Trap treatment.

Indeed VEGF blockade may affect vessel survival through multiple actions, as correctly pointed out by the Reviewer, and it is certainly possible that VEGF blockade may destroy unstable vessels by cutting off flow, or direct apoptosis of endothelial cells, or other mechanisms still. However, we have adopted a rigorous definition of vascular stabilization as the ability of newly induced vessels to persist independently of VEGF stimulation, which is a therapeutically relevant property defining the minimum necessary duration of VEGF delivery to achieve a persistent increase in vascularity. Therefore, stable angiogenesis can only be quantified as the net increase in vessel length density that resists VEGF-blockade and the rationale for selecting VEGF-Trap to test stabilization of newly induced vasculature rests on the fact that it potently abrogates VEGF signaling *in vivo*, regardless of what specific downstream processes are affected.

2. For the injected myoblasts: did the authors observe differences between the Tibialis and Gastrocnemius muscle? From the images provided in the figures it is not clear which muscle was used.

We have now clarified the structure of in vivo experiments entailing muscle tissue samples in the Methods (page 23, first paragraph). We observed no differences in the results obtained in the two muscle locations. Therefore, all experiments have been carried out in equal numbers of each and results have been pooled together.

3a. Figure 1: this figure needs to be clarified. Please provide a macroscopic image or illustration showing where the myoblasts were injected, and which muscle was analyzed (Tibialis / Gastro). It is difficult to see the skeletal muscle organization on the images provided; how are the sections projected (transverse/longitudinal)? In a normal histological section, skeletal muscle fibres are surrounded by several capillaries and only a few small arterioles and venules. Can the authors indicate these vascular compartments in their sections. Please provide an image of a normal, uninjected control, and images of the "site remote of myoblast implementation, the area with VLD 15 mm/mm2).

We have clarified the topographical interpretation of the images shown in Fig. 1 in the associated legend: fibers are mostly caught in longitudinal orientation, but, depending on the specific muscle bundles, sometimes partial transverse profiles are also visible. Areas of implantation have been selected based on X-gal staining of serial sections to reveal the β-gal expressing myoblasts (page 25, first paragraph). Areas of effect comprise capillaries both wrapping around large pre-existing fibers and in interstitial locations, where small fibers were generated by fusion of the implanted myoblasts with each other, as already described in earlier work with this platform (see, for example, Springer et al. Dev. Cell 2000; Springer et al. Mol. Ther. 2003). The induced vasculature was represented almost exclusively by capillary networks, as correctly pointed out by the Reviewer, and no smooth muscle-coated arterioles or venules are visible in the images shown in Fig. 1 for the Vlow and Vmed conditions, but only capillaries associated with NG2+ pericytes. The only smooth muscle-coated structures in implantation sites are the angioma-like aberrant vessels induced in the high VEGF conditions, as we previously showed (e.g. Ozawa JCI 2004).

Lastly, we have now clarified in the Results (page 6, first paragraph) that the images shown for the control conditions indeed represent sites remote from myoblast implantation and were taken from sections actually devoid of injection sites, as determined by X-gal staining of adjacent serial sections.

3b. Can the authors provide evidence showing that VEGF-trap actually reached the implanted myoblasts, and reduced VEGF availability and/or signalling in endothelial cells? Are the effects comparable between the arteriolar and venular part of the vascular bed investigated? VEGF has been linked to specifying arterial identity in growing capillaries - is this inhibited upon VEGF-trap in this setting and would it influence the "stabilization" measurements?

We have now clarified the established efficacy of VEGF-Trap to abrogate VEGF signaling in vivo in the Results (page 5, first paragraph). VEGF-Trap, which is a chimeric receptor-body and therefore has the pharmacodynamic behavior of an antibody, has been extensively used and characterized in animal experiments and human clinical trials (as detailed also in the response to point 1. above), showing its ability to access even difficult compartments like tumors, characterized by high oncotic pressure. Furthermore, being delivered systemically, it has direct access to all endothelial cells reached by the general circulation and the data in Fig. EV1 show that essentially all vessels induced by all VEGF doses in our experiments were functionally perfused. Lastly, the effective vascular regression induced by the treatment, further confirms that the drug reached its targets.

We have not investigated the arterio-venous specification of the vessels induced by the different VEGF doses. However, while this is certainly an interesting aspect of the effects of VEGF blockade, our results are unlikely to be affected by these considerations because: 1) induced vessels consistently displayed the morphology of microvascular networks of pericyte-covered capillaries, except for aberrant angioma-like structures, which never stabilized; and 2) vessel persistence was quantified as vessel length density, regardless of the morphology of visible vascular structures in any analyzed field. On the other hand, the possible influence of different VEGF doses on the arterio-venous specification of newly induced vascular structures is certainly an interesting subject that should be addressed specifically in future work.

4. Why do low and medium VEGF levels induce pericyte recruitment and high VEGF levels VSMC? It is not clear why recruitment of VSMC, as seen in high VEGF, didn't result in a stabilized vessel. In the context of vessel stabilization, what is the functional difference between pericytes and VSMC recruitment? Skeletal muscle arterioles and venules are surrounded by VSMC and they are usually considered "stable". Can the authors clarify this issue?

The Reviewer raises interesting points, which we have addressed in the discussion (pages 16, last paragraph and 17, second paragraph). The origin of pericytes and smooth muscle cells on normal and aberrant vessels is not completely understood. However, previous results obtained with the same myoblast-based platform show that, while low VEGF levels retain pericytes close to remodeling vessels, high levels cause pericytes to disappear from initially activated vessels (Gianni-Barrera et al. Angiogenesis 2013), consistently with the anti-pericyte effect of VEGF (Greenberg et al. Nature

2008), while the smooth muscle coating appears later upon remodeling to angioma-like structures. Whether this represents selective recruitment from the arterial or venous side of the network, in situ differentiation of progenitors, or even the result of endothelial-to-mesenchymal transition is debated and should be addressed by specific future studies.

Indeed normal arterioles and venules are coated by smooth muscle and are stable. However, the aberrant angioma-like structures induced by high VEGF are neither arteries nor veins, rather they are aberrant structures, continuously growing at a slow rate and behave functionally like arterio-venous shunts (Zacchigna et al. Hum. Gene Ther. 2007). Further, pericytes and smooth muscle cells are functionally different, as pericytes are known to provide mainly molecular cross-talk regulating endothelial function rather than mechanical support that is the main function of smooth muscle cells.

5a. In some settings like the nervous system and sprouting blood vessels, Semaphorin-3A is considered to act as a repulsive guidance cue involving Neuropilin-1 and Plexin receptors. What turns Sema3A into an attractive agent with respect to recruitment of neuropilin-1 positive bone marrow derived cells?
5b. What percentage of the total monocytes/macrophage population expresses neuropilin-1?
5c. Neuropilin-1 is also expressed by growing sprouts and arterioles (it is considered an arterial marker). Is there any evidence for an endothelial cell-autonomous, Sema3A-neuropilin-1 autocrine loop contributing to the monocyte, or pericyte recruitment upon grafting of the VEGF producing myoblasts?
5d. In the collateral arteriogenesis setting, VEGF has been shown to promote influx of macrophages/monocytes into growing and expanding collateral arteries. In the myoblast setting, high VEGF levels associate with reduced recruitment of monocytes; can the authors explain this difference?

These are all interesting points, although they may be somewhat peripheral to the focus of this manuscript, rather branching out into the field of Sema3A and Nrp1 basic biology.

5a. Indeed Sema3A acts as a repulsive cue also for endothelial cells by signaling through Nrp/Plexin co-receptor complexes. On the other hand, NEM attraction is mediated by the specific interaction of Sema3A with the Nrp/VEGF-R complex and recent evidence shows that the attractive function of Sema3A on monocytes requires activation of VEGF-Receptor in the holoreceptor complex with Nrp1 and Plexin (Casazza et al. Cancer Cell 2013). While the underlying molecular mechanism for the attractive action of Sema3A on Nrp1-expressing monocytes has not been completely elucidated, the chemotactic action of Sema3A on BM CD11b+ cells has been demonstrated to specifically require binding with Nrp1 by Zacchigna et al. (JCI 2008), as well as by our data in Fig. 4, showing the effects of treatment with an antibody specifically blocking the binding of Nrp1 with Sema3A.

5b. Carrer et al. (Cancer Res. 2012) have also found that Nrp1 is expressed on about 5% of total BM monocytes, of which about 1 in 5 (or 1% of total BM monocytes) are NEM and are attracted by Sema3A and VEGF. In contrast, the same group (Zacchigna et al. JCI 2008) has shown that most monocytes recruited to muscles by expression of VEGF or Sema3A are NEM, and our data presented here in Fig. EV2 confirm that essentially all monocytes present in the areas of implantation of VEGF-expressing myoblasts are NEM, showing that they are specifically recruited from the circulation.

5c. Sema3A has indeed been shown to participate in endothelial cell-autonomous signaling, which mediates the inhibitory effects of Sema3A on VEGF-induced angiogenesis, both through competition for the shared receptor Nrp1 and by inducing the production of the decoy VEGF-Receptor soluble Flt1 by endothelium. However, no such autocrine loop is known to affect monocyte or pericyte recruitment. We have discussed these mechanisms at pages 19-20.

5d. Lastly, in collateral arteriogenesis VEGF recruits monocytes/macrophages through MCP1 and other cytokines, whereas here we found that the recruitment of the specific Nrp1-expressing subpopulation of monocytes in the setting of microvascular angiogenesis requires endogenous

Sema3A. Therefore, different monocyte populations, recruited by different chemokynes, are involved in the two processes.

6. Figure 3: can you indicate the site where the myoblasts were implanted?

We have now clarified in the Methods (page 25, first paragraph) that all analyses shown in the Results were carried out exclusively in the areas of myoblast implantation, as determined by X-gal staining on adjacent serial sections.

7a. Figure E2: the variance in the Sema3a measurements is too large to make any statement. This experiment needs to be repeated (compare the error bar in VEGF low with VEGF high).

In the new Figure S2 actual data points are now provided. Clearly there is variability in the Vlow condition, but the other values are quite homogeneous and the data show that Vhigh myoblasts do not produce less Sema3A than Vlow or Vmed, which is the only conclusion drawn from these results to exclude that the reduced expression of Sema3A observed in vivo could be explained by a VEGF-dependent regulation of its production from the implanted myoblasts. Further, the new *in situ* hybridization data in Figure 2C-D show that greater than 90% of the Sema3A-expressing cells in areas of effect *in vivo* are actually endothelial, further corroborating this conclusion.

7b. To provide direct evidence showing that the Sema3A - monocyte axis is essential, the authors should consider depleting the mouse macrophage / monocyte population pharmacologically or genetically and analyse vessel stabilization upon low VEGF treatment.

We agree with the Reviewer that an experiment of VEGF blockade with the VEGF-Trap while blocking NEM recruitment with the anti-NP1 antibody would have been ideal. Unfortunately, we could not perform this experiment due to the refusal of the permission from the pharmaceutical company holding the rights to the VEGF-Trap (and providing it for this project) to combine it with the anti-Np1 antibody, whose rights are held by a competing pharmaceutical company. However, the results of the blocking experiments with the anti-Np1 antibody in Figure 4, together with the other data on the role of TGF-β1 signaling (Figures 5 and 6) and acceleration of stabilization after treatment with recombinant Sema3A (Figure 7), provide a consistent and homogeneous body of evidence for the conclusions of the manuscript, namely that VEGF dose-dependently impairs vascular stabilization by inhibiting the endothelial Sema3A/NEM/TGF-β1 axis.

8. With respect to stabilization: what is causing the switch between low and high dose VEGF. Do these results imply that the amount of VEGF-R2 phosphorylation is the key regulator? Can the authors provide data indicating how this switch is mechanistically regulated?

We have now provided further mechanistic evidence (Figure 6D-E) showing that expression of Sema3A by activated endothelial cells and NEM recruitment require TGF-β1 signaling and now provide further discussion of the proposed model for the switch between low and high VEGF (pages 18-19). These new data corroborate the proposed model shown in Figure 8, supporting the *in vivo* function of a positive feedback loop between TGF-β1, endothelial Sema3A, NEM recruitment with associated TGF-β1 production and further Sema3A expression. As VEGF does not affect Sema3A production directly at low levels, this feedback loop is active in conditions of low VEGF, leading to the maintenance of vessel-stabilizing signals and endothelial SMAD2/3 activation. On the other hand, at high levels, VEGF has a direct inhibitory effect on Sema3A production by endothelial cells, thereby breaking the feedback loop and impairing NEM-dependent vessel stabilization. The further mechanism by which VEGF dose inhibits Sema3A expression by endothelium is an interesting open question and a role for the degree of VEGF-R2 phosphorylation is a plausible hypothesis to be investigated in future studies.

9a. For the rescue experiments: the authors should show that the injected Sema3A protein activated Neuropilin-1 signalling in the monocyte population.

We have not directly shown Nrp1 activation in the recruited NEM. However, the results of the *in vivo* blocking experiments in Figure 4 prove functionally that NEM recruitment requires the specific

binding of Sema3A and Nrp1. On the other hand, the requirement for Nrp1 to mediate monocyte attraction by recombinant Sema3A has already been shown by Zacchigna et al. (JCI 2008).

9b. In the same line: where are the neuropilin-1 receptors expressed in this setting (endothelial cells, monocytes,VSMC/pericytes/muscle tissue) - is it possible that Sema3A directly acts on pericytes or smooth muscle cells?

Neuropilin1 is indeed expressed both on monocytes, endothelium and pericytes. However, it is unlikely that Sema3A may promote vascular stabilization by acting directly on pericytes, as it has been shown by others that Sema3A binding to Nrp1 is repulsive for pericytes (Jurisic et al. Circ. Res. 2012). Therefore a direct effect on pericytes should cause pericyte loss in conditions of low VEGF (high Sema3A) and their retention in conditions of high VEGF (low Sema3A), whereas our data indicate the opposite effects.

9c. Surprisingly, Sema3A treatment didn't affect the amount of vascular growth (Figure 8). However, monocytes are considered to stimulate angiogenesis, promote fusion between angiogenic sprouts and arteriogenesis. How do the authors explain that on the one hand Sema3A promotes influx of monocytes, but on the other hand doesn't affect angiogenesis?

Sema3A has been reported to actually have anti-angiogenic effects in several settings (see also response to point 5c above) and we have interpreted the lack of negative effects we observed on vascular growth in the Discussion (pages 19-20). On the other hand, it is true that some monocyte populations (which are not Nrp1-positive) have a pro-angiogenic effect. However, essentially all monocytes recruited to VEGF-expressing muscles in our experiments are Nrp1-positive NEM (Figure EV2) and NEM are a specific population that does not produce pro-angiogenic factors (Carrer et al. Cancer Res. 2012), nor stimulate endothelial proliferation (Zacchigna et al. JCI 2008), but rather have a pro-maturation and pro-stabilization function. We have now better clarified the relationship between NEM and other monocyte populations in the Introduction (page 4, first paragraph).

10. The authors may want to test their findings in an ischemia model - for example the femoral artery ligation assay. If a cocktail of low VEGF and Sema3A indeed makes functional perfused vessels that can carry blood flow to the compromised regions, then the findings may have a broad impact.

We have now addressed the potential therapeutic relevance of our findings in a clinically relevant model of adenoviral VEGF gene therapy in immunocompetent animals (Fig. 7D-F) and we found that indeed local Sema3A treatment with only 2 doses injected during the first week is sufficient to increase NEM recruitment and stabilize the induced vasculature, completely preventing its regression by 3 weeks that is otherwise observed in this transient expression system, thereby confirming the results obtained in the immunodeficient SCID model. Preclinical development in relevant ischemia models, testing the ability of Sema3A treatment to provide long-term therapeutic benefit with short-term VEGF delivery, will be the subject of ad hoc future studies.

2nd Editorial Decision 06 July 2015

Thank you for the submission of your revised manuscript to EMBO Molecular Medicine. We have now received the enclosed reports from the referees that were asked to re-assess it. As you will see the reviewers are now fully supportive. Please note that Reviewer #3 was not available to re-evaluate your revised manuscript and I had therefore asked Reviewer 1 to perform this in his/her lieu. I am pleased to inform you that we will be able to accept your manuscript pending the following final minor requirements:

1) Please provide a new manuscript without the red lettering indicating the changes, as this is no longer needed.
2) Every published paper now includes a 'Synopsis' to further enhance discoverability. Synopses are displayed on the journal webpage and are freely accessible to all readers. They include a short standfirst as well as 2-5 one sentence bullet points that summarise the paper. Please provide the synopsis including the short list of bullet points that summarise the key NEW findings. The bullet points should be designed to be complementary to the abstract - i.e. not repeat the same text. We encourage inclusion of key acronyms and quantitative information. Please use the passive voice. Please attach this information in a separate file or send them by email, we will incorporate it accordingly.

I look forward to seeing a revised form of your manuscript as soon as possible and in any case, within two weeks.

***** Reviewer's comments *****

Referee #1 (Remarks):

I believe that the authors made a commendable job in responding to the Reviewers' comments, and I am certainly satisfied of how they responded to my requests.

Referee #2 (Remarks):

The authors have answered my comments and made changes to the manuscript accordingly. The manuscript is highly improved during the reviewing period with clarification of a number of issues. It is important to illuminate the mechanisms of stabilization of VEGF-induced neavasculature, thus the manuscript should be published in EMM.
